# A Novel Tyrosinase Gene Plays a Potential Role in Modification the Shell Organic Matrix of the Triangle Mussel *Hyriopsis cumingii*

**DOI:** 10.3389/fphys.2020.00100

**Published:** 2020-02-19

**Authors:** Gang Ren, Chao Chen, Yefei Jin, Genfang Zhang, Yiwei Hu, Wenying Shen

**Affiliations:** ^1^School of Life Sciences, Shaoxing University, Shaoxing, China; ^2^College of Life Sciences, Zhejiang University, Hangzhou, China; ^3^College of Agriculture and Bioengineering, Jinhua Polytechnic, Jinhua, China

**Keywords:** tyrosinase, *Hyriopsis cumingii*, shell formation, shell regeneration, RNAi

## Abstract

Although tyrosinases have been speculated to participate in the shell formation of mollusks, there is still a lack of experimental evidence to support this assumption. In this study, a novel tyrosinase designated *HcTyr2* was isolated and characterized from the freshwater mussel *Hyriopsis cumingii*. The change in *HcTyr2* mRNA expression during the process of embryonic development was detected by real-time quantitative PCR. The result showed that the expression of *HcTyr2* mRNA was significantly upregulated at the stages of gastrulae and unmatured glochidia (*P* < 0.05), suggesting that this gene might fundamentally participate in the biogenesis and growth of the initial shell. Meanwhile, the upregulation of *HcTyr2* mRNA at the stages of shell regeneration 24 h and 9 days after shell notching in the mantle edge (*P* < 0.05) implied that it might play an important role in shell periostracum and nacre formation by mediating the cross-linking of quinoproteins to promote the maturity of organic matrix. Additionally, the knockdown of *HcTyr2* mRNA by RNA interference resulted in not only the suppression of periostracum growth but also structural disorder of nacre aragonite tablets, as detected by scanning electron microscopy. These results suggested that *HcTyr2* might regulate the growth of shell by its oxidative ability to transform soluble matrix proteins into insoluble matrix proteins, then promoting the maturity of the shell organic framework in *H*. *cumingii*. In general, our results suggested the importance of *HcTyr2* in the shell formation and regeneration of *H*. *cumingii*.

## Introduction

Tyrosinase is a member of the type-3 copper protein superfamily and catalyzes the hydroxylation of monophenols and the oxidation of o-diphenols to o-quinones (Garcia-Borron and Solano, 2002). Data from transcriptome and genomics has shown that tyrosinase genes have expanded substantially in the pearl oyster of *Pinctada* sp. (Du et al., 2017), the Pacific oyster *Crassostrea gigas* (Yu et al., 2014; Huang et al., 2017), and the Zhikong scallop *Chlamys farreri* (Li et al., 2017), implying their versatile functions in these species (Aguilera et al., 2014). In detail, tyrosinase acts not only as a key enzyme to synthesize melanin, participating in the color pattern of shell (Feng et al., 2015; Sun et al., 2016; Yu et al., 2017, 2018), but also as a member of the prophenoloxidase (proPO) cascades in the innate immune system of mollusks, playing important roles in melanization and clot formation during wound healing (Zhou et al., 2012; Allam and Raftos, 2015; Wang et al., 2018).

Recent research has shown that tyrosinases are strongly expressed in the pallial and edge of the mantle, which are the main biomineralization tissues of Mollusca (Zhang et al., 2006a; Nagai et al., 2007; Yu et al., 2014; Huening et al., 2016; Yang et al., 2017), and tyrosinase proteins have also been detected in the proteome of the shell prismatic layer and/or nacreous layer (Marie et al., 2012; Liao et al., 2015; Mann et al., 2018). These results implied the crucial functions of tyrosinases in periostracum sclerotization and the cross-linking and maturation of shell matrix proteins (Zhang et al., 2012; Aguilera et al., 2014; Huening et al., 2016; Du et al., 2017).

*Hyriopsis cumingii* is the important freshwater mussel, producing the largest amounts of cultured pearls in the world. Transcriptomic data of this species showed that at least six tyrosinase transcripts existed in the mantle tissue, implying that the expansion of this gene family has also occurred during the evolution of this species (Aguilera et al., 2014). Recently, the differential expression patterns of two novel tyrosinase genes, *HcTyr* and *HcTyp-1*, were reported in the mantle of *H. cumingii*, suggesting their potential different roles in nacre coloration (Chen et al., 2017). In addition, our results for the transcriptome showed thirty unigenes annotated as tyrosinase genes expressed in the pearl sac of *H. cumingii* (GenBank BioProject No. PRJNA556395), but their biological functions need be further studied.

In this study, a novel tyrosinase designated *HcTyr2* was identified in the freshwater mussel *H. cumingii*. The spatial and temporal expression patterns *HcTyr2* mRNA were analyzed by real-time quantitative PCR. The change of its mRNA expression after shell notching was also detected. Meanwhile, the function of *HcTyr2* in shell growth and nacre crystal structure was also studied by the method of RNA inference. The aim of this work is to provide more experimental evidence to uncover the function of the tyrosinase gene in the shell formation of *H. cumingii*.

## Materials and Methods

### Mussels

Mussels used in this study were obtained from a seed multiplication farm (Jinhua Jewel Pearl Institute, Jinhua, China). Two-year-old mussels with a shell length of 110 ± 10 mm were used in the cDNA cloning and analysis of the tissue expression pattern of *HcTyr2* mRNA and of the expression pattern of *HcTyr2* mRNA in the mantle edge after shell notching. One-year old mussels with a shell length of 60 ± 10 mm were used in the experiment on RNA inference (RNAi). Embryos at the cleavage stage, larvae at the stages of blastocyst, gastrulae, unmatured glochidia, and matured glochidia, and juvenile mussels 10 days after dropping off the host fish were collected to detect the temporal expression pattern of *HcTyr2* mRNA.

### Identification of Molecular Characterization of *HcTyr2*

#### Amplification and Verification of the *HcTyr2* cDNA Sequence

To verify the sequence of the unigene annotated as tyrosinase (*HcTyr2*) from the transcriptome of mantle, 2.0 μg of total RNA from the mantle edge was reversely transcribed with a PrimeScript^®^ reverse transcriptase kit (TaKaRa, Dalian, China) and primed with Oligo(dT)_18_. The specific primers for the amplification of the *HcTyr2* cDNA fragment, including the 5′-end, middle, and 3′-end of fragments, were designed according to the unigene sequence (Table 1). The PCR product was purified, cloned, and sequenced using the methods described in Ren et al. (2014).

**TABLE 1:** Primers used in the present study.

**Primer name**	**Sequence 5′ → 3′**	**Sequence information**
Tyr5-F	CGGTTCGAGGCGTGAGATTC	cDNA sequence verification
Tyr5-R	GCAACGGGTTCCATCACAG	cDNA sequence verification
TyrM-F	GCTGAATACGGCAGCTCATG	cDNA sequence verification
TyrM-R	TGCCAGACCAGTGCCAGATA	cDNA sequence verification
Tyr3-F	TGTGGACGAGTCTGTCAACCG	cDNA sequence verification
Tyr3-R	GCCATTTATGTAGCCAAGATAAGAA	cDNA sequence verification
RT-HcTyr2-F	AACGGTGATGGTGTCGTGGTA	qRT-PCR
RT-HcTyr2-R	CTGTTGGCATCAGAATGTCGG	qRT-PCR
T7-HcTyr2-F	TAATACGACTCACTATAGGGA CGGTGATGGTGTCGTGGTA	RNAi
T7-HcTyr2-R	TAATACGACTCACTATAGGGC CTCCTGCCTGAACCTCCTA	RNAi
T7-GFP-F	TAATACGACTCACTATAGGGCGAC GTAAACGGCCACAAGT	RNAi
T7-GFP-R	TAATACGACTCACTATAGGGCTT GTACAGCTCGTCCATGC	RNAi
RT-TYR2RNAi-F1	CCCATCTTCAACCCCACAAG	RNAi
RT-TYR2RNAi-R1	GAAACGACGGGGTACACGG	RNAi

#### Bioinformatics Analysis

Multiple alignments of amino acid sequences were performed using ClustalX 1.81 software^1^ and edited with GeneDoc software^2^. Prediction of functional domain was performed using the Simple Modular Architecture Research Tool (SMART)^3^. The sequences used to construct the evolutional relationships of Bivalvia tyrosinase proteins were downloaded from the reference of Aguilera et al. (2014) and transcriptome data from *H. cumingii* tissue of pearl sac (GenBank BioProject No. PRJNA556395). The potential deduced proteins annotated as tyrosinases were identified by SMART searches. All the sequences are listed in the Supplementary Dataset File S1. The phylogenetic tree of Bivalvia tyrosinases was constructed with Mega 7.0 software using the Neighbor-Joining method with 1,000 bootstrap replicates (Kumar et al., 2016).

### Temporal and Spatial Expression Analysis

#### Sample Collection

Ten tissues, namely hepatopancreas (HP), gill (GL), adductor muscle (AM), foot (FT), gonad (GN), hemolymph (HM), pearl sac (PS), mantle center (MC), mantle edge (ME), and outer epithelium of the posterior mantle pallial (OpMP), were isolated from 2-year-old mussels (*n* = 6). The samples were frozen in liquid nitrogen and then stored at −80°C. About 100 mg of embryos at the cleavage stage and larvae at the blastocyst, gastrulae, and unmatured glochidia stages were isolated from the gill cavities of different fertilized mussels (*n* = 3) during the spawning season (May 2017). Each sample of matured glochidia was collected immediately from a basin when the glochidia dropped from 5 to 7 individuals of parasitic host fish *Pelteobagrus fulvidraco* (*n* = 3). Juvenile mussels, cultivated for 10 days after glochidia had dropped from the host fish, were collected from the sediments of different nursery tanks (*n* = 3).

#### Real-Time Quantitative PCR Assay

Quantitative real-time PCR (qRT-PCR) assay was performed to detect the relative expression level of *HcTyr2* mRNA using the method described in Ren et al. (2014). The gene-specific primers (RT-HcTyr2F and RT-HcTyr2R) for qRT-PCR were designed according to the complete *HcTyr2* cDNA sequence (Table 1). *Elongation factor 1-alpha* (*EF1-α*) was used as an endogenous reference gene for calibration with the primers described in Chen et al. (2017). Real-time RCR was performed in the following program: denaturation at 95°C for 1 min, 40 cycles of 95°C for 15 s, 56°C for 15 s, and 72°C for 15 s, following the determination of the melting curve with 0.5°C increments from 50°C to 95°C. The relative mRNA expression levels (N) were calculated using the following formula *N* = 2^(Ct*EF*1α−Ct*HcTyr*2)^.

#### Expression of *HcTyr2* mRNA Under the Stimulation of Shell Notching

A fan-shaped notch with length and width of 15 × 5 mm was cut in the posterior of the left shell of 2-year-old mussels, and a total of 40 operated-upon mussels were cultured in a pond with water temperature fluctuating between 26 and 30°C. The mantle edge adjacent to the shell notch at the left side of each operated-upon mussel was isolated, and the mantle edge at the right side of the same mussel was also sampled as control. Four operated-upon mussels were sacrificed at hours 0, 12, 24, and 36, and days 2, 3, 4, 6, 9, and 12 after shell notching, respectively. The relative expression level of *HcTyr2* mRNA (N) in the mantle edge after shell notching was analyzed using qRT-PCR as mentioned above and calculated using the formula *N* = 2^Ct^*^*EF*1α^*^(left side)–Ct^*^*TcTyr*2^*^(left side)^/ 2^Ct^*^*EF*1α^*^(right side)–Ct^*^*TcTyr*2^*^(right side)^.

#### RNA Interference Experiment

An RNAi experiment was performed according to Suzuki et al. (2009), with some modification. The first strand of cDNA was reverse-transcribed from the total RNA of the mantle edge. The specific DNA fragment was amplified by Ex Taq^®^ DNA Polymerase (TaKaRa) using gene-specific primers containing a T7 promoter, T7-HcTyr2F, and T7-HcTyr2R. The PCR product was purified, cloned, and sequenced using the method described in Ren et al. (2014), and the positive plasmid was extracted as the DNA template to synthesize HcTyr2 dsRNA using an AxyPrep^TM^ Plasmid Miniprep Kit (Axygen, Hangzhou, China). *HcTyr2* dsRNA was synthesized and purified according to the method described in Suzuki et al. (2009). GFP dsRNA was synthesized as a negative control using the plasmid of pEGFP-C1 (Clontech, Palo Alto, CA, United States).

The injection dose of dsRNA was calculated according to the weight of 1-year old mussels to be a final concentration of 1 μg/g body weight (*n* = 8). In the experimental group, *HcTyr2* dsRNA was injected in the front adductor muscle (*n* = 8). The same dose of PBS or *GFP* dsRNA in PBS, separately, was injected and treated as control groups (*n* = 8 each). Meanwhile, a triangular notch with a width of 3 mm and depth of 4 mm was cut in the posterior of the left shell to detect the inhibition of periostracum growth after *HcTyr2* RNAi. The operated-upon mussels were cultured in a pond with a greenhouse, which had a temperature of 29 ± 2°C during the whole experimental period. At day 3 post-injection, the same dose of *HcTyr2* dsRNA, PBS, or *GFP* dsRNA was re-injected into the posterior adductor muscle of each mussel. The mussels were sacrificed and the right-side mantle edge was sampled at day 5 post initial injection to detect the change of *HcTyr2* mRNA expression after RNAi using the qRT-PCR method described above and the specific primers of RT-TYR2 and RT-TYR2. The shell at the posterior of the right-side shell was cut off, and its inner nacre was washed ultrasonically with absolute ethanol and 75% alcohol. The nacre pieces were then etched in Na_2_EDTA solution (0.5 M) for 8 s, rinsed with deionized water immediately, and then dried in air. The inner nacreous surface was sputter-coated with gold and visualized using a JSM-6360LV scanning electron microscope (SEM, JEOL, Tokyo, Japan) to investigate the morphology of the growing nacre tablets. The rate of periostracum regeneration (R) at day 5 post-injection was calculated with the formula *R* = height of new growth periostracum (H)/depth of notch (D) in the left shell. H and D were the vertical distance from the top of the triangular notch to the ventral bottom of new-growth periostracum and notch, respectively.

#### Statistical Analysis

One-way ANOVA followed by Duncan’s or Tukey’s *post hoc* test was performed to test the differences of the relative expression level of *HcTyr2* mRNA in the temporal and spatial expression analysis, the shell notching experiment, the RNAi experiment, and the differences in the rate of periostracum regeneration in the RNAi experiment. Differences were considered statistically significant at *P* < 0.05 and very significant at *P* < 0.01. All the statistics were performed with SPSS 25.0 software (SPSS Inc., Chicago, IL, United States).

## Results

### Sequence Characteristics and Evolution Relationship Analysis

**FIGURE 1:**
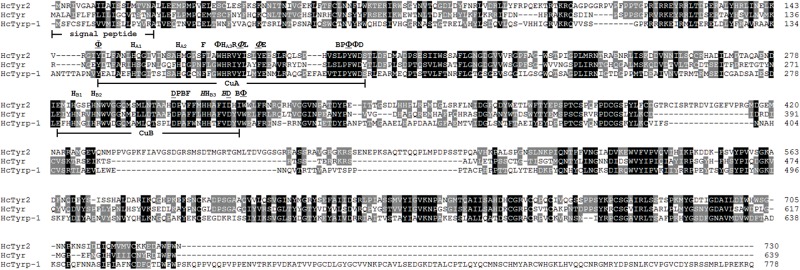
Multiple alignment of three amino acid sequences of tyrosinases from *H. cumingii*. Identical residues are highlighted in black, and similar residues in 80 and 60% identifications are highlighted in gray and light gray, respectively. The GenBank accession numbers of HcTyr and HcTyrp-1 are APC92581.1 and APC92582.1, respectively. Φ mean aromatic residues, F, Y or W. H_A1_, H_A2_, H_A3_ and H_B1_, H_B2_, H_B3_ are conserved histidine residues in the Cu A and Cu B motifs, respectively. An underlined residue name means the residue is a conserved residue that is important for maintaining the three-dimensional structure of tyrosinase. An italicized residue name means that the residue may be involved in the affinity switch from o-diphenols to indoles.

**FIGURE 2:**
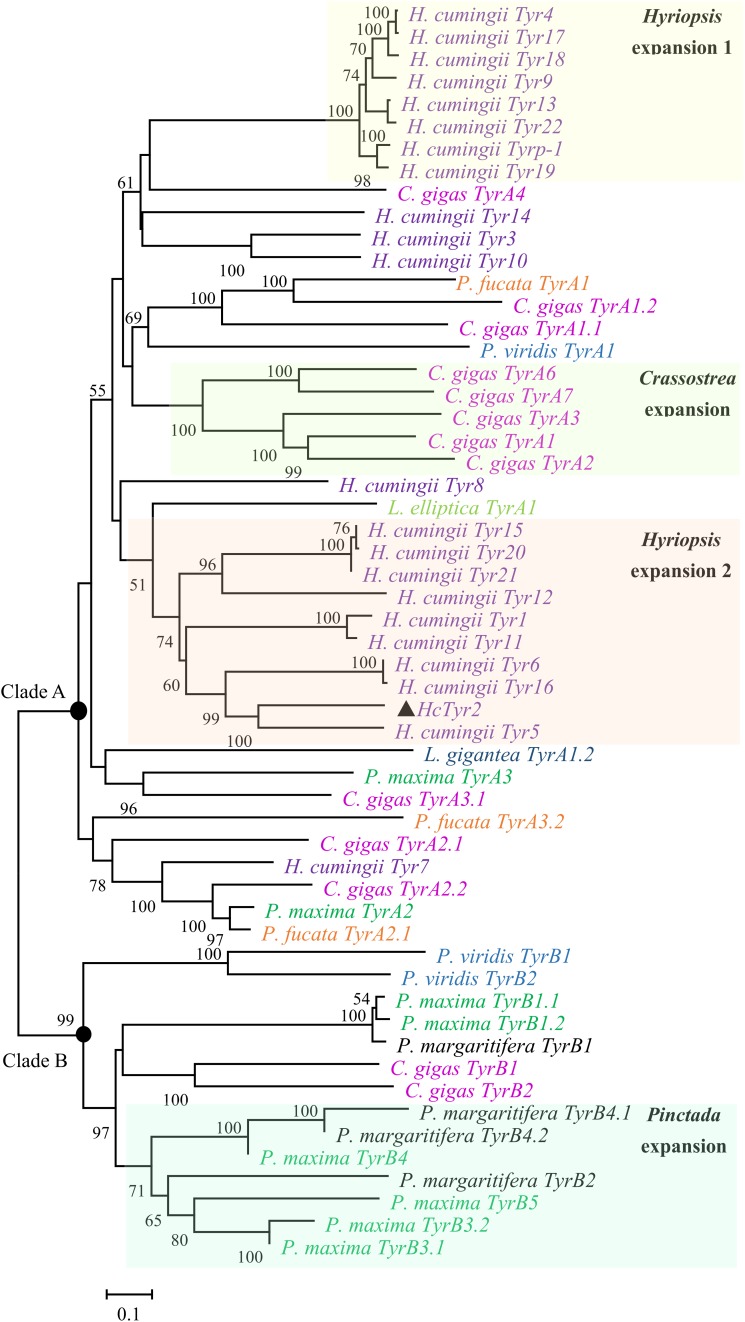
Phylogenetic analysis of Bivalvia tyrosinase proteins by the Neighbor-Joining method. The evolutionary distances were computed using the Poisson correction method. The tyrosinase protein sequences are selected from Aguilera et al. (2014). The percentage of replicate trees in which the associated taxa clustered together in the bootstrap test (10,000 replicates) are shown next to the branches.

The sequence of the unigene named the *HcTyr2* gene was confirmed by cDNA cloning and sequencing and included a 97-bp 5′-UTR, a 2193-bp ORF, and a 742-bp 3′-UTR (GenBank accession no. MN508358). The deduced amino acid sequence with a length of 730 aa contained a signal peptide and a tyrosinase domain (Figure 1). In the tyrosinase domain, there were two His-based regions named Cu A and Cu B, which contained the conserved H-X(8)-H-X(8)-H motif and H-X(3)-Hx(21)-H motif, respectively. The phylogenetic tree constructed by the Neighbor-Joining method showed that the Bivalvia tyrosinase proteins divided into two clades, an ancestral form (clade A) and a diverged form (clade B) (Figure 2). Nevertheless, a total number of 23 *H. cumingii* tyrosinases including *HcTyr2* clustered in clade A, and these transcripts had undergone large expansions in this species like *Pinctada* spp. and *C. gigas* (Aguilera et al., 2014).

### Spatial and Temporal Expression Pattern of *HcTyr2* mRNA

**FIGURE 3:**
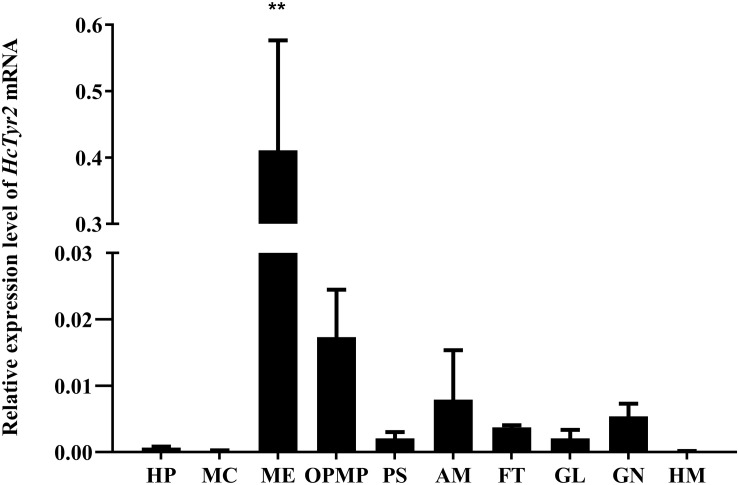
Relative expression levels of *HcTyr2* mRNA in tissues of *H. cumingii* as analyzed by qRT-PCR. *EF1-α* used as a reference gene. HP, hepatopancreas; MC, mantle center; ME, mantle edge; OpMP, outer epithelium of the posterior mantle pallial; PS, pearl sac; AM, adductor muscle; FT, foot; GL, gill; GN, gonad; HM, hemolymph. Each bar represents mean ±SEM (*n* = 6). Statistical significance is tested using one-way ANOVA followed by Duncan test (**P < 0.01).

**FIGURE 4:**
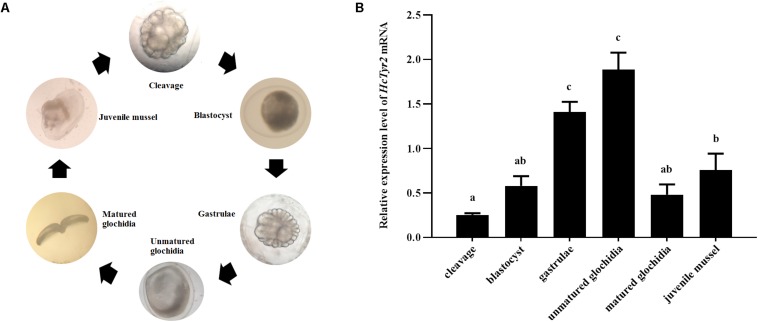
**(A)** Embryonic developmental process of *H. cumingii*. **(B)** Relative expression levels of *HcTyr2* mRNA at different developmental stages of *H. cumingii* as detected by qRT-PCR. *EF1-α* used as a reference gene. Statistical significance is tested using one-way ANOVA followed by Tukey test. Means with different superscript letters are significantly different at *P* < 0.05.

The spatial expression profile detected by qRT-PCR showed that *HcTyr2* mRNA was detected in OPMP, PS, AM, FT, GL, and GN but was mainly expressed in the biomineralization tissue, the mantle edge (*P* < 0.01) in the 2-year old mussel (Figure 3). The embryonic development of *H. cumingii* had six stages, including cleavage, blastocyst, gastrulae, unmatured glochidia, matured glochidia, and juvenile mussel (Figure 4A). During this course, the expression of *HcTyr2* mRNA was significantly upregulated in the early stages of cleavage to gastrulae and reached its highest value at the stage of unmatured glochidia, but was remarkably downregulated in the later stages of matured glochidia and juvenile mussel (*P* < 0.05) (Figure 4B).

### The Effects of Shell Notching on *HcTyr2* mRNA Expression

**FIGURE 5:**
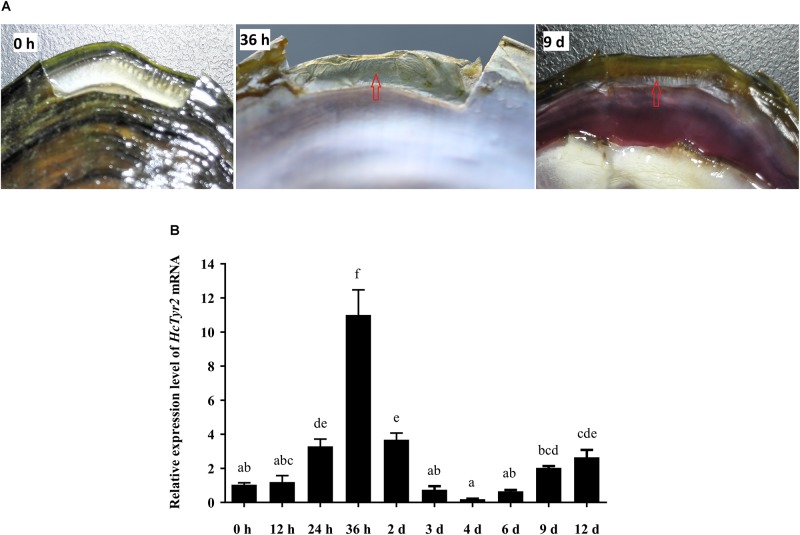
Effect of shell notching on the expression of *HcTyr2* mRNA. **(A)** Shell regeneration status of 2-year-old mussel at 6 h, 36 h, and 9 days after shell notching. The red arrows indicate regenerated periostracum (36 h) and nacre (9 days). **(B)** Changes in relative expression levels of *HcTyr2* mRNA in mantle edge after shell notching. *EF1-α* used as a reference gene. Statistical significance is tested using one-way ANOVA followed by Duncan test. Means with different superscript letters are significantly different at *P* < 0.05.

Twenty-four hours after shell notching, the regeneration of shell periostracum was begun at the notching site in the shell edge, and a newly formed periostracum could be clearly observed at 36 h after shell notching (Figure 5A). Nine days after shell notching, the periostracum was completely repaired, and nacreous deposition could be found at the position of the repaired inner shell (Figure 5A). Meanwhile, when compared with the control group (0 h after shell notching), the expression level of *HcTyr2* mRNA in the mantle edge was significantly increased (10.5-fold) at 36 h after shell notching (*P* < 0.01) and gradually decreased until day 4 after shell notching but re-increased at day 6 to day 12 after shell notching (Figure 5B).

### The Effects of RNA Interference on *HcTyr2* mRNA Expression and Shell Biomineralization

**FIGURE 6:**
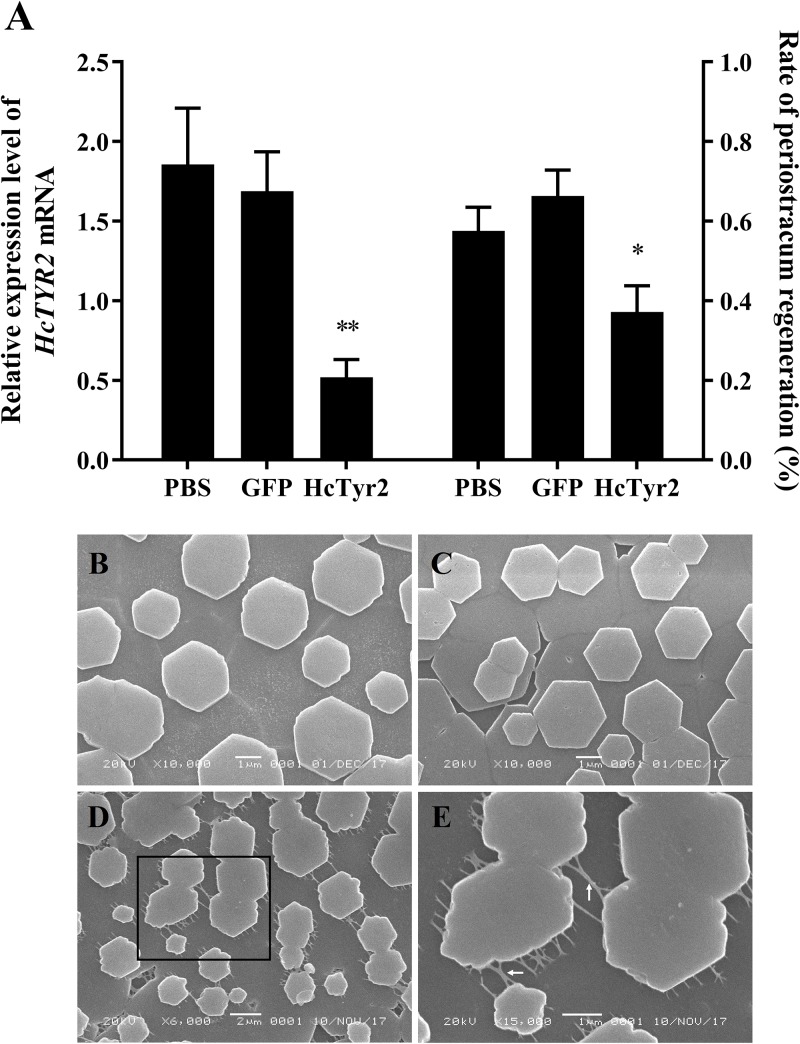
Knockdown of the *HcTyr2* mRNA by RNA interference. **(A)** Changes in relative expression levels of *HcTyr2* mRNA in mantle edge and rate of periostracum regeneration after RNA interference. *EF1-α* used as a reference gene. Statistical significance of relative expression level and rate of periostracum regeneration were tested using one-way ANOVA followed by Duncan test (^∗^*P* < 0.05, ^∗∗^*P* < 0.01). **(B–E)** Scanning electron microscopy (SEM) images of nacre tablet microstructures on the surface of new-growth nacreous layer of 1-year-old mussels after injection of PBS **(B)**, *GFP* dsRNA **(C)**, and *HcTyr2* dsRNA **(D)**. **(E)** is a high magnification view of the region (black box) in **(D)**. The white arrows in **(E)** indicate filamentous inter-laminar organic matrix (ILOM).

Five days after injection, the growth of regenerated periostracum at the notching site of the left shell was significantly inhibited in the dsHcTyr-injection group (Figure 6A, *P* < 0.05). Meanwhile, the SEM microstructure of the nacreous-layer surface showed that the newborn aragonite tablets had a typical hexagonal shape (Figures 6B,C) in the control groups (PBS- and dsGFP-injection groups). Nevertheless, the newborn tablets had an irregular hexagonal shape in the experimental group, and the inter-laminar organic matrix (ILOM) connecting the crystal basement was exposed and in a filamentous form (Figures 6D,E). The relative expression levels of *HcTyr2* mRNA in the mantle edge of the experimental group (dsHcTyr*2*-injection group) were remarkably suppressed by approximately 70% when compared to those in the control groups (Figure 6A, *P* < 0.01).

## Discussion

The species-specific expansion of tyrosinase genes had been reported by Aguilera et al. (2014) in the pearl mussel *H*. *cumingii*. In this study, a novel tyrosinase named *HcTyr2* was identified in this species. The deduced protein sequence of HcTyr2 contained the important tyrosinase domain with two copper-binding sites of CuA and CuB, in which the conserved H-X(8)-H-X(8)-H motif and H-X(3)-Hx(21)-H motif were also found, respectively (Garcia-Borron and Solano, 2002; Figure 1).

Recent genome and transcriptome data showed that the tyrosinase gene family had undergone large expansions in many species, including the pearl oysters *Pinctada* sp. and the Pacific oyster *Crassostrea gigas* (Aguilera et al., 2014). Most of the tyrosinase genes in these bivalves were more highly expressed in the mantle, especially at the distal mantle edge, the tissue responsible for shell biomineralization (Zhang et al., 2006a; Marie et al., 2012; Huening et al., 2016). Recently, Chen et al. (2017) reported that two tyrosinase genes, *HcTyr* and *HcTyp-1*, were highly expressed in the dorsal epithelial cells of the mantle pallial of *H. cumingii*. Nevertheless, *HcTyr* and *HcTyp-1* had markedly different expression profiles in the mussels with purple or white nacre, suggesting their important roles in periostracum and nacre coloration (Chen et al., 2017). In this study, the phylogenetic analysis showed that the tyrosinase genes underwent species-specific gene expansion twice in *H. cumingii* (Figure 2). *HcTyr2* was diverged with *H. cumingii Tyr1* (*HcTyr*) and *H. cumingii Tyrp-1* (*HcTyrp-1*) (Chen et al., 2017), implying the potential possibility of their functional difference. The relatively high expression of *HcTyr2* detected in mantle edge implied its potential role in shell biomineralization (Figure 3). However, no significant difference in the relative expression level of *HcTyr2* mRNA was found between the mantle pallial of the mussels with purple and white nacre or between pearl sac-producing purple and white pearls (Supplementary Figure S1), suggesting that the function of *HcTyr2* might not be directly related to the nacre coloration. Additionally, the highest expression level of *HcTyr2* was detected at the embryonic developmental stages of gastrulae and unmatured glochidia in *H. cumingii* (Figure 4). At the stage of gastrulae, the embryo developed the original shell gland and began to form an original shell; then, the shell secretion was sped up at the stage of unmatured glochidia (Wang et al., 2007a). During the period of parasitizing in host fish *P. fulvidraco*, the larvae developed from the unmatured glochidia to matured glochidia, but their shell length and height increased slightly (Wang et al., 2007b). However, the growth of shell length and height resumed quickly once juvenile mussels released from the fish to begin the free-living stage. Thus, the expression profile of *HcTyr2* might coincide with shell formation and growth during embryonic development. This result indicated that *HcTyr2* might participate fundamentally in the biogenesis and growth of the initial shell from gastrulae to juvenile mussel. The mRNA expression pattern of specific tyrosinases in parallel with shell matrix deposition in early larval shell biogenesis was also found in the Pacific oyster *C. gigas* (Huan et al., 2013) and the Mediterranean mussel *Mytilus galloprovincialis* (Miglioli et al., 2019).

Tyrosinases catalyze the oxidation of both monophenols and o-diphenols into reactive o-quinones (Garcia-Borron and Solano, 2002). Data from shell proteomics showed that tyrosinases were incorporated into the shell matrices in the prismatic layer (Nagai et al., 2007; Liao et al., 2015; Mann et al., 2018), nacre layer (Mann et al., 2018), and even the whole shell (Zhang et al., 2012; Du et al., 2017) of molluscan species. Recent research showed that tyrosinases participated in periostracum sclerotization and the cross-linking and maturation of shell matrix proteins by catalyzing the quinoprotein oxidation (Zhang et al., 2012; Aguilera et al., 2014; Du et al., 2017). Meanwhile, the upregulation of tyrosinase genes in mantle after shell damage indicated that tyrosinases participated in initial shell regeneration, especially the formation of organic sheet in periostracum (Wang et al., 2013; Huening et al., 2016). The formation of periostracum is the first step of shell growth and regeneration, providing an organic template for initial CaCO_3_ crystal deposition (Marin et al., 2008). However, there is still a lack of direct proof to illuminate the role of tyrosinase in shell biomineralization. In this study, the significant upregulation of *HcTyr2* mRNA at the initial stage of periostracum and nacre regeneration (24 h and 9 days after shell notching) in mantle edge (Figure 5) implied its important role in shell regeneration by mediating the cross-linking of quinoproteins to promote the growth and subsequently the maturity of shell periostracum and nacre (Du et al., 2017). This result was further confirmed by the RNAi experiment, in which the regeneration of periostracum was obviously inhibited in the dsHcTyr*2*-injection group (Figure 6A). In addition, inhibition of tyrosinase activity by kojic acid reduced the growth-slowing of *H*. *cumingii* periostracum after shell notching (Supplementary Figure S2). An organic framework of chitins and insoluble matrix proteins was made of a continuous organic “lace-like” or “foam-like” network to provide the surface for the crystallization of individual aragonite tablets in the nacre layer (Rousseau et al., 2005). Bedouet et al. (2007) proposed that the oxidation of water-soluble matrices was a leading process in the transformation of soluble matrix proteins into insoluble matrices to form the fiber network during nacre growth. Quinone-tanned proteins contained a Gly/Tyr-rich region, and Prismalin-14 and KRMP were considered to be involved in the cross-linkage of the matrix framework of *P. fucata* with the prismatic layer (Suzuki et al., 2004; Zhang et al., 2006b). However, the molecular basis of oxidative reaction in the nacre layer has not been investigated in detail. Research showed that the organic matrix around aragonite tablets of *H. cumingii* nacre included inter-laminar organic matrix (ILOM) and inter-crystalline organic matrix (Song et al., 2018). In this study, ILOM in the control groups was recovered by the growing aragonite tablets; however, knockdown of *HcTyr2* by RNAi mainly reduced the disorder of ILOM and aragonite tablets (Figures 6C,D). This result implied that the suppression of *HcTyr2* expression might have decreased the oxidative ability of organic matrix and impeded the transformation of soluble matrix proteins into insoluble matrix proteins and the formation the organic framework, thus generating the disorder in the structure of nacre ILOM and aragonite tablets. In summary, our results supported the important function of *HcTyr2* in the modification of organic matrices during the shell formation of the pearl mussel *H*. *cumingii*.

## Data Availability Statement

The raw data supporting the conclusions of this article will be made available by the authors, without undue reservation, to any qualified researcher.

## Author Contributions

GR and WS designed the experiments, analyzed the data, and wrote the manuscript. GR, CC, and YJ carried out the experiments. YH wrote the manuscript. GZ contributed the materials. All authors carried out the revision of the manuscript and gave final approval for publication.

## Conflict of Interest

The authors declare that the research was conducted in the absence of any commercial or financial relationships that could be construed as a potential conflict of interest.

## Footnotes

1 http://www.clustal.org/

2 http://nrbsc.org/gfx/genedoc

3 http://smart.embl-heidelberg.de/
