# Supplementary material for: A Novel Tyrosinase Gene Plays a Potential Role in Modification the Shell Organic Matrix of the Triangle Mussel Hyriopsis cumingii
Source: Front Physiol. 2020 Feb 19;11:100. doi: 10.3389/fphys.2020.00100 (PMC7045039; doi:10.3389/fphys.2020.00100)
Supplement: Supplementary file 1 [file Data_Sheet_1.docx]

**SUPPLEMENTARY MATERIAL**

**MATERIALS AND METHODS**

**Tyrosinase activity assays**

Kojic acid with the concentration of 6 mmol L^-1^ was injected into the adductor muscle of 2-years old mussels (n = 12) at a final dose of 1 μl/g body weight (n = 12), and re-injection every 3 days until the mussels were sacrificed at 15 days after injection. Sodium phosphate buffer (PBS, 0.1 mol L^-1^ pH 6.8) was injected into other 12 mussels as control. The tyrosinase activity was measured according to the method by Raftos et al. (2007) with slight modifications. 20 μl homogenate from mantle edge (100 mg) was incubated with 200 μl L-DOPA (5 mmol L^-1^) for 2 hours at 36 °C. Tyrosinase activity was expressed as the change in optical density (OD) at 490 nm (OD490) of 1 ug protein in 2 hours of reaction (U mg prot^-1^).

**RESULTS**


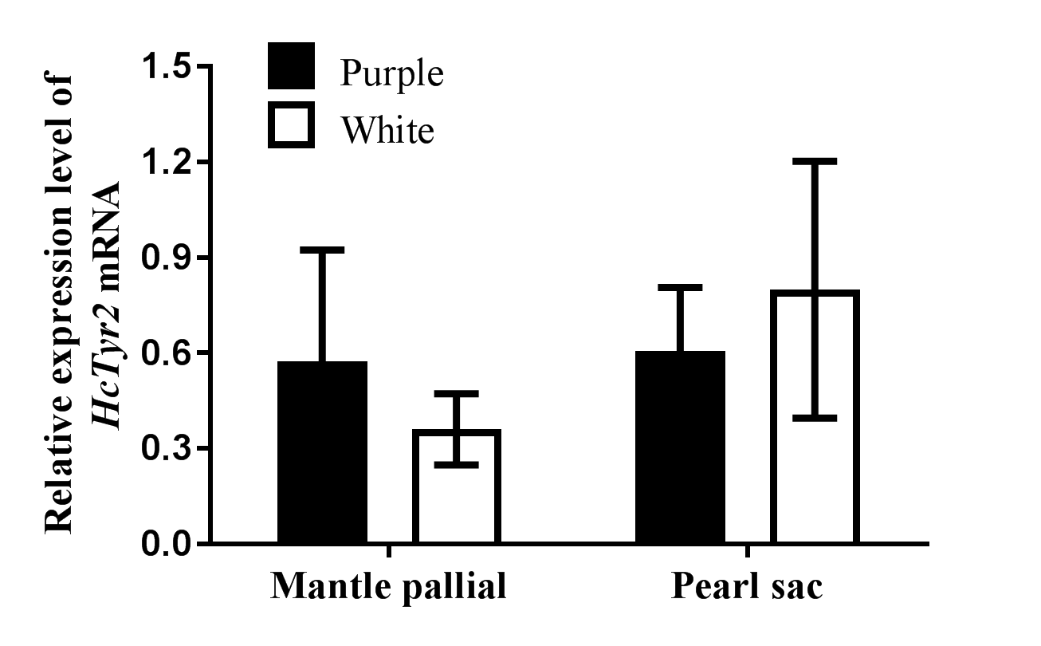


**Figure S1** The relative expression level of *HcTyr2* mRNA detected by qPCR in tissues of mantle pallial and pearl sac producing purple and white nacre or pearl.


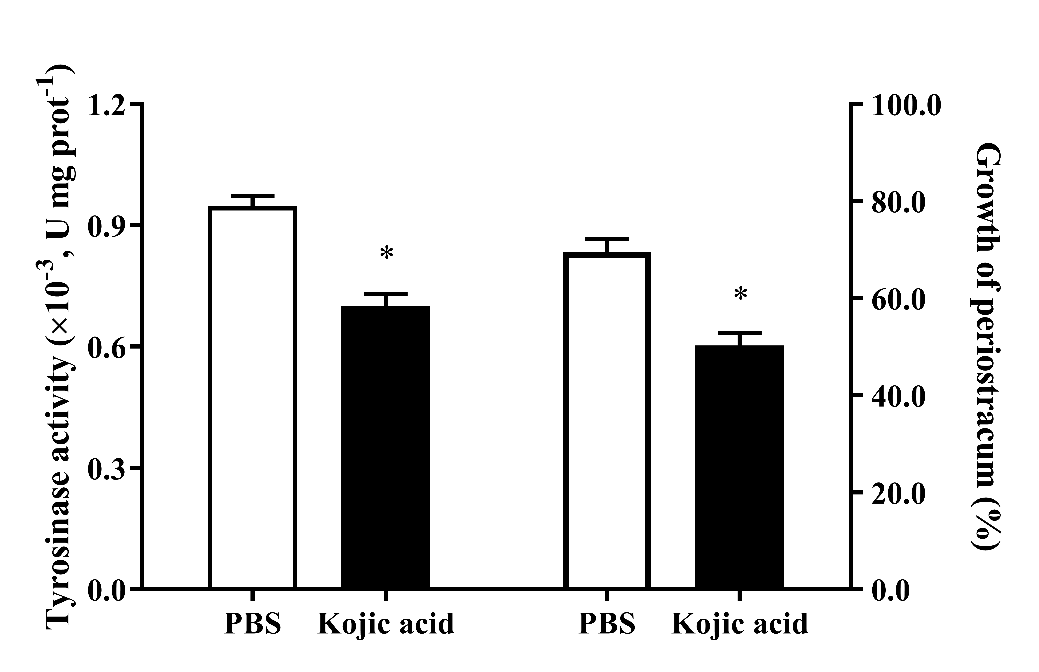


**Figure S2** Effects of kojic acid on tyrosinase activity in mantle edge and the growth of shell periostracum after 15 days of shell notching.

**REFERENCES**

Raftos, DA., Aladaileh, S., Rodney, P., and Nair, S. V. (2007). Characterization of phenoloxidase activity in Sydney rock oysters (*Saccostrea glomerata*). *Comp. Biochem. Phys. B*. 148, 470–480. doi:10.1016/j.cbpb.2007.07.089

**Supplementary dataset file S1.** Tyrosinase protein sequences used in the phylogenetic analysis.

> *Hyriopsis cumingii* Tyr1 (HcTyr, APC92581.1, complete)

MGRNTYSDLKYYAENKCSQRVTSMALAIFLFFLILIGCVTSTNAYLEFLPMPQEMTSCFNYFHQKCNLTNTVGHSLNRHCVNSYYFRSEKIKWEWTNLTDFDMHYLKFLKRRIFDNHFRAKRQSDINPSPPSGFRVRKEYRRLTDSERTAYHSVLNVMKRNGEYDTFARIHSGPNLGQFHDGPNFLGWHRIYLAYFEEAVRRYDNSLSLPFWDYTLDFPLSDPTQSVLWSATFLGNGDGVVWSGPFSGWVVNGSPLIRNTGHQGALMSKQDVDTVLTRCDTSEITFPRETGTYNLEIYHNRVHNWVGGNMELLDTAAFDPAFFLHHAFVDYVWELFRLRQLTVCGINPEANYPNVVGDHAPQREMHAFPQHRASDGYANYWTDSWYTYEMSPSCSRVRPDCGSLYLKCDIDRDICVSKTRSEIKTSRTSSTERRKRSALVLETPSSCTGTISGTMQNTYLINGNNDIDSWVYIPIQIAYIRPSGYHFHSYPIQDGKVQYVQDVYSPFLYPNLHSYVKSEDLKAYPNCGLDPSGAGQIYVRSDGLTYYGTYVDYVLSDVRQPYGSTVVYIGVKNPGSGISEAILSAHDKCGRPCSVTCAKPNTDPPSYKRCSGVIRLSARLPLMYGSNYGDAVLSAWDPLGMGPEFNGIHVIIICNYRDIWPW

>*H. cumingii* Tyrp-1 (Hc Tyrp-1, APC92582.1, complete)

MSFCSFLSVVMISLLPYVRATVEQTNVPDELTWNFYVAQHKTSRLNTPSNAIQSWCTNVYKWQHDSIVHGGRNASTGTRELVHYLSDQVLQSVHHIGRVKRQVVSSGPKRRRKEIRMLTERELDLYFRAVRAAKANTTTAPNVYEALAEFHTGITSISAHGGCNFFGWHRVYLLMYENMLRAQGPEFAEVTIPYWDSRLEARMEQPTSTVLFTNRFLGTGSGEVSGGILGSGWQTSAGPLIRNIGTDGPPMTDEAIVNVTRMTRMSEICGADSAIESDLEFHHNGIHRWVDGQMAMLQTSPLDPAFWNHHTFVDYVWEAFRINSRRNGVNIETDYPANPTTMGAAELHAPDAALGFAEMTVIDGLSNTFTAEIYEYDPPPTCSVQNPDCGSKYLKCVIFSNNAHCVSRTLAEVLEWENNQVTRTTVAPVTSPPPTACPHPPTTQLYTEHDKPYQNHYCLNGKSDIRQWVYIPVKIIYRRPPEYTSYGSYPIYNGKISKFYDIYAPNVYSNVYQHLKNGQPAKYEKCSEGDKRISSIYIKSVGLSYDGTYKEYAIVDRRIAITVSTAYIAVKNPAKESSLALLQATDSCGRACRPVCKVRNSNIYRPCSGAVRLTSAFPRHYGSDFGNAVMDVWDFTADKSCPQFNNASIFIAFNCDFQTDWFWPSKQPPVQQPVPPENVTRKPVDKATVVPGCDLGYGCVVNKPCAVLSEDGKDTALCPTLQYQCMNSCHMYARCWHGKLHVQQCNRGMRYDPSNLKCVPGVCDYSRSSMRLPREKRQ

>HcTyr2 (complete)

MNRIVGAAILAISSLMTVNALLEEMPMPVELESCLESFKSKNNITNIVGEKLFTFCLNNFLWKTERIRWSGYNVTQGDLDYFNRLVDRLLYFRPQEKTRTKRQAGPGGRPVPFPPTGPRIRREYRRLTDPERALYHRLINELKVRGIYDLFANLHQGIVINSAHMGPSFLAWHRVYISLYEESLRQLSPHVSLPYWDSTLDFDMADPSESIIWSSAFLGNGDGVVVRGPFAGWSTPIGPLMRNTRAENRLISKDDVNNILSQCRHADILMPTAQAENDIEMIHGSPHNWVGGQMSMLNTAAHDPVFFMHHAFIDHIWWLFRNRQRHVCGVNPATDYPEITTGSDLHHPLRPMDGLSRFLNIDGFQDYWETLWFTYEPSPTCSPQFPDCGSPYLFCDGTRCISRTRDVIGEFVPRGMDGEMNARRAMGEVQNMPPVGPKFIAVGSDGRSMSDTMGRTGMLTDVGGSGRRASSRAVGRGKRSSENEPKSAQTTQQPLMPDPSSTPQAVHKPALSPGNSLNKPIQNTFFVNGIADVKEWVFVPVQVIHIKPKDDKFSVYPVVSGKADFNQDFYSISSHALDARIKQGHPKEFSNCKADPSGAAQVVLQSVGINYMGRYSEFAIIDSRLPIASSMVYIGVKNPANGMTQAIISAHDHCGRVCQPQCIVQGSSPPSYKPCSGAIRLSSTSPKMYGDTIGDAILDIWHWSGNNPKNSIDDIQMVMVCKKEDAWPWN

> *H. cumingii* Tyr3 (complete)

MTPWRLVLLAALVNEVVCMIEEIPLPPDIQECLDQVHAKTNLTEVVGENVVWRCVHGSLWKKAKENPNNQLSDKALKWFATLINKSVHKSDLQGRRSRVRRQTARLRVRREYRMLSDTERNNFHQALNMLKADTSIPPNRFDSLGLLHQTQGIRSHDGPNFLGWHRLFLIMAENALREKIPTVTIPYWDSTLDAALPDPRASVFFSPEFMGDAIGAVDTGPFSGWRTPIGPLIRHFGQDGYLMNWTSIRDVFTRSRLEEITAPHAVSKYNIEQHHAGPHLWVGGYMSPQALAGYDPIFYLHHSFVDLIWELFRNIQRRGGVDPTTDYPTNITIEGQGGQDQNGFNTLTNRDALSDYYTTTIYSYQLPPTCTTEQPDCGSPYLHCDTAGSSPRCVSVTIFDPKPSDLMHENGLPMNGSPRRNRVTKRSANQTGNGHRNTLEEGTLCKGKNWDQQYTNNYFIDGLIDKKLWAYIPVGIIFKNDDHTFLTHQNSSVLAVYSKCIDGVPISPHVTIESNGLNYEGMYKEITPVDKDLPIFSTTTFIGVKSPDTNGSEVLISAYDACGRVCQAFCRDTSSKSASLDMPCSGAIRITRDSPLLYGRSASEVAENLFKTPEAGAFPEFQRNEMFLQFYCKQEDWPWKSKFSHL

> *H. cumingii* Tyr4 (complete)

MSFCSFLSVLMISLLPYVRATVEETNVPDELTWNFYVAQHKTSRLNTPSNAIQSWCTNVYKWQHDSIVHGGRNASTGTRQLVNYFSDQVHLSVRRTGHAKRQVAPSGPKRRRKEIRMLTEKELDLYFRAVRAAKTNTTTAPNVYEALAEFHTGITSISAHGGCNFFGWHRVFLLMYENMLRDQGPEFAEVTIPYWDSRLEARMDQPTSTVLFTDRFLGTGSGEATGGILGSGWTTSAGPLVRNIGTDGPPMTDEAIVNVTRMTRMREICGADSSIESDLEFHHNGIHRWVDGQMAMLQTSALDPAFWSHHTFIDFVWEAFRINSQRNGVNIETDYPANPTTMGAAELHAPDAALGFAEMTVIDGLSNTFTSQIYEYDPPPTCTVQNPDCGSKYLKCVVFRDNAHCVSRTLAEVIQWENDQTRFTTVAPTAPIVPTPPPSGCSNPSPPPTHTQEDKPYQNHYCLNGKSDIRQWVYIPVKIIYRRPPEYKSYGSYPIYNGKSSKTNDIYAPSAYSHVYQYLKSGEPAKYEKCVEGDKRISSVYIKSVGLNYDGIYKEYAIVDRRIAITVSTAYIAVKNPATESSLALLQATDSCGRVCRPVCKVPNSNIYRPCTGAVRLTSAFPRHYGTDFGNAVMDIWDFTAEKSCPQFNTENIFIAFYCDFQTDWFWPTKEPPTQQPVPPGHVIHKPGKAYGTGMGQNPQVGAAPITDNAAAVPGCDLGYGCIVSKPCAVLSQEGKDTGLCSTLQYECTNSCHMYAKCWYGKLYVQQCNRGMRYDPSSLKCVPGVCDYSHSSMGLPRG

> *H. cumingii* Tyr5 (complete)

MTGKLVRATFVALIGVVLVSGLIEEIPMPPELQSCLKDFHTKTNITDTVGEKIFSYCLNSFLWKTETTNWGEFNITQADLEYFHRLVDQLITRHVPSRGKRQALGPVLFPPTGFRIRREYRRLNGMERDAFHRAVIELKRRGEYDLFARMHQGIVIQSAHNGPNFLGWHRVYITMYEEALRRIIPMVVLPYWDSTLDFHMDEPTESVIWTPLFMGDGSGVVNTGPFTSWSTPIGPLRRNIGVQSRLISRQNVIDILSRCRHAEITFPAALPQFNLEFIHGGPHGWVGGHMAGLNTAAFDPIFFLHHAFIDYIWQLFRNHQRIVCGVNPATDYPPATGLQVPQRPMDAFPRFTNIDGYADYWEMFWFNYEPSPTCTMSLQCGSPWLICQGGFCISRAAEVSGLPAARGFAGEMEARRGREETASMPDVGQRFIMPPSESRTHEFRSRTRGSLTDVEGSEMRAMIGRVKRAAGVAPLSDNMTIAEKPGDISLSTKSYKAGTGLVTPVQNSFFINGEADMKLWAFVPIQVIYMKPSNEKFPVHPVYNGQPDLNQEFYSINYRDMDKQTRDGHPKEFTNCKRDPSGAGQILIQSNGMNYVSRYIEYAIVDSRLPIGSSMVYMGVKNPVNETVEAIISAHDRCGRMCEPRCLIHGSNPPRYRPCSGAIRISSASPKMYGNTFGEAIMDVWHWSGSDPRNSINNIQMVMICNNDEKWPWN

> *H. cumingii* Tyr6 (complete)

MFLLLVTAAAFMSVAYALMEEMPMPPELLSCLQGFQVKTNITSTVGEEMFTFCLNKFLWKANSIKWSGYNVTEADLRFISSITTSLFHRRRTKRQTGPGNAPGTGFRVRREYRMLSDAERNAFHTALNEMKRGGQYDTFARLHAGIVVNSAHGGPNFFGWHRVYLSLFEEALRRINQNISLPYWDSTMDFDMANPSESILWSSRFLGNGDGVVTSGPFANWNAGGAPLTRNIGGTSRLFSKQDINNILSRCRTREISERTALPQYSVEFIHGGPHNWVGGQMSGLNTAAFEPTFFLLHAFVDYIWELFRIRQRQFCNVNPSVDYPTATNLHASNRAMDGFSGYRNIDGYANYWTQLWYNYESAPTCSRSNPSCGSPYLLCDQAREVCVSRTREAIGEDVPRGFAAAESSSRSRMQEAMIDVGPRFSAPPSDGRSVDVQGGILGGASTVQAELLGASMGPRFNPPPSDGRTADAGFAAFSGINSDKVHEAAAETIVPQPSIIPGGNLIRPIQNTFLLNGVADTSKWSYVPVQIIHSRPNGEIYSSFPINGGIVNLQQDVFSPEQYPQLKAFINPGHPSGYEHCKSDPSGAAQIYVQSDGMNYNGRYADYAVIDARLPVGSGMVYMGIKQPSNAMSDVIFSAHDRCGRMCQPQCLVNGTRPPIYKPCSGAVRFTPSTPKMYGNTFGDAVMSVWNMINGDPQNYGGSVPLVMACNYKDSWPWDHCNQGSGQ

> *H. cumingii* Tyr7 (complete)

MPSIAVFLILLFAVIPYSDLQAQVTSNRSLDPSFLRWLNSLFYLPPEGELRVRKEYRMLTDIERNNFHHAMRLLKMDTSVPPNKFDALASLHHMNTAEAAHGGPNFLGWHRVYLVLCENALREKIANVTIPYWDNTLEEALPDPRQSILFSPLFMGSASGQVVTGPFSYWSTVGYGQLARDVGNSRRLMNPGDLTAIFSRRLIADISNPNAPESSNLEELHNDVHVYVGEQMSRIESASYDPLFYIHHAFIDCVWEEFRNLQRLSGVDPARDFPRIVGEQAHQPLAAMGLGRLLVIDGINNIFTQRIYRCESRPACIMNSNTCGSPYLRCDWTRQRCMPLIMSSGPGMFARSQWTFPGFFAG

> *H. cumingii* Tyr8 (patial)

GARVRKEIRMLTDSERNDFFRAVNMLKNDKSLEPNVYDNFTDLHLINILRSHFGPAFVAWHRVYLYMFETLLRTKVPGVTLPYWASALDREMHDPTQSVMWADEFLGNGFGEPITGPFSNWTKINPIQYFERQIGTSGELFSYDGLQRVLSKQRNEQIFQPTAPFGDSLEDQHGAVHQWIGGSMRNLVSAPHDPLFFLHHAFVDLIWESFRIRQFQSSLDPTRDYPWDRNNGRLPSEHAPESDMGFSFVRVSNIQGFGTGFSNLVQYDPIPSCTALRPRCGSRFLRCDTNATRPRCVSRAISDVPGLTNYVNILSGMGRPDREMCPIPETDRPAQNNFAINGVVDVNNWVYVPVRIISKRPPSFRRFSYLKSSGQQSATSIPESNQATYKDCKIEDTVAGKIKVVSIGLNYIGQYEEFAIVDNRQEIAVSSISIAVRKPLYQHTETLVSAFDKCGRVCRPFYSRTDKGDDEPFSGAILLTSSGTVQYQSSASESIKTAWTVGSDDACPVFSIKSPYITFYCDYRGIWPWEMYKNDSGVSKISIPTRFRNVENQGNLDEKGQLAVINERKDMPSTVQIDTAKASAGDSSTSYVQTLAAQEKPNPCNNVYPTSPVDMALCKKQIQGTLQIKNAR

> *H. cumingii* Tyr9 (complete)

MIWVFSLTVIMISLVPYVKATVEQTDVPEELTWNFYVAQHKTSRLNTPSNAIQSWCTNVYKWQHDNIVHGGRNASTGTRQLVNYFSDQVHLSVRRTGHAKRQVAPSGPKRRRKEIRMLTEKELDLYFRAVRAAKTNTTTAPNVYEALAEFHTGITSISAHGGCNFFGWHRVFLLMYENMLRDQGPEFAEVTIPYWDSRLEARMDQPTSTVLFTDRFLGTGSGEATGGILGSGWTTSAGPLVRNIGTDGPPMTDEAIVNVTRMTRMREICGADSSIESDLEFHHNGIHRWVDGQMAMLQTSALDPAFWSHHTFIDFVWEAFRINSQRNGVNIETDYPANPTTMGAAELHAPDAALGFAEMTVIDGLSNTFTAEIYEYDPPPTCSVQNPDCGSKYLKCVIFSNNAHCVSRTLAEVLEWENNQVTRTTVAPTAPIVPTLPPSGCSNPSPPPTHTQEDKPYQNHYCLNGKSDIRQWVYIPVKIIYRRPPEYTSYGSYPIYNGKISKFYDIYAPNVYSNVYQHLKNGQPAKYEKCSEGDKRISSIYIKSVGLSYDGTYKEYAIVDRRIAITVSTAYIAVKNPAMESTLALIQATDSCGRVCRPVCKAPNSNIYRPCSGAVRLTSAIPRHYGTDFGNAVMDVWDFTTDKTCPQVKYENILIAFNCDFQTDWFWPTKQPPAHRPLPSGHKIHKPIHGPKLVKG

> *H. cumingii* Tyr10 (complete)

MSENAMREKIPTVTIPYWDSTLDAKLPDPRASILFSPEFMGTGKGIVNTGPSRHWRTPWGPLIRNFGQDGVLMNWTSIRDVFTRRRLSEITMPNAEAKYNFEDIHGEPHLWVGGHMSPQALAGYDPIFFLHHSFIDLLWEIFRMLQRMRGVDPTKDYPNNTVPEGHGLNDPSGFEGLTNEQALSDYYTSNIYTYELPPSCSRERPNCGSPYLRCDTSVAKPSCVSLTIFDTQPADFMLENEFSPNGGVSRLRENRNHGGNGRVDHVQEITRDTNFLWNVFGGNRRGRRAAQNNGTESMALSKLTSIFETSYSSVSENQNLQCQPNDWNEQYVNNFNINGLTDRKQWVYIPVGIIYRKDPERLGFNYHSHLQVYSKCKRGIYINPHVSIESIGLNYMGIYKEVIPINKDTPVFSTSSFIGVKVPHGNVTDVLLVAYDVCGRVCQPYCF

> *H. cumingii* Tyr11 (complete)

MHYLKFLKRRIFDNHFRAKRQSDINPSPPSGFRVRKEYRRLTDSERTAYHSVLNVMKRNGEYDTFARIHSGPNLGQFHDGPNFLGWHRIYLAYFEEAVRRYDNSLSLPFWDYTLDFPLSDPTQSVLWSATFLGNGDGVVWSGPFSGWVVNGSPLIRNTGHQGALMSKQDVDTVLTRCDTSEITFPRETGTYNLEIYHNRVHNWVGGNMELLDTAAFDPAFFLHHAFVDYVWELFRLRQLTVCGINPEANYPNVVGDHAPQREMHAFPQHRASDGYANYWTDSWYTYEMSPSCSRVRPDCGSLYLKCDIDRDICVSKTRSEIKTSRTSSTERRKRSALVLETPSSCTGTISGTMQNTYLINGNNDIDSWVYIPIQIAYIRPSGYHFHSYPIQDGKVQYVQDVYSPFLYPNLHSYVKSEDLKAYPNCGLDPSGAGQIYVRSDGLTYYGTYVDYVLSDVRQPYGSTVVYIGVKNPGSGISEAILSAHDKCGRPCSVTCDQTKYRSAILQEMFWCHSSVRASSANVWFKLWRCSAQCLGSAWNGT

> *H. cumingii* Tyr12 (complete)

MSPITLLLFVTALPLLVTADLQYMSWPDHLFSCLDSFQKVTDITEEVGEHIFRHCIDVQNIKNSKLSWTWVNITGVGDQPRAKRQATTPVTSPPSGYRTRKEYRTLTDDERNRFHAALNAMRRTGEFQRFASYHRNIPGRNVLQEFHNGPAFLGWHRVYLARFEEALRRYDNTVSLPYWNSSLDFYMTGDPANSVLWSDLFLGTRSGFVTSGPFAGWPGGANSILTRDVMLNRRGLLISNDDVNNVLRLCSTADVSFPPRRPLNSIIEFHHNRPHTFVGGNTGDMSTLPNAAFDPAFFLHHAYVDYIWELFRTRQRSACNVDPTTDYPNTVPIQSPNSFMWGNELSAYRNIDGFADYWTRNWYGYQPTPTCSSSNPDCGSRYLRCDTTRNTCVSRSASEVSSTTGKRRKRQSRVPFSDVNDIYDNVYTPKQNVYSVDGVCDTKLWVYIPVQIVHIRPKGYTFSSFPVTAGQLDASFDVYHAPSESYLYNYSQPGNPKEYQQCQKYESGAKPIYVTSDGISYYGTYTDYAIVDGRLPVQSSVVYIGVKKPSGTPTEAIFSARDICGCVCKPQCLVRNSSPPVYKPCSGAIKLTDSATNMYASTIYEATMMDWEISFGSIRSKKPDIAIVFTCTFDNEWPWIQPQSTQNEGQRRGHHVQTPGGLRTRYR

> *H. cumingii* Tyr13 (complete)

MIWFISLPVVMISLVPYVKATVEQTDVPEELAWNFYVAQKKTSTSNTPANAIQSWCTNVYKWQHDNIVHGGRNVTTGTRQLVNYFSDQVHLSVRRTGLVKRQVAPSGPKRRRKEIRMLSEKELDLYFRAVRAAKANTTTAPNVYEALAEFHTGITSISAHGGCNFFGWHRVYLLMYENMLRAQGPEFAEVTIPYWDSRLEARMEQPTSTVLFTNRFLGTGSGEVSGGILGSGWQTSAGPLIRNIGTDGPPMTDEAIVNVTRMTRMSEICGADSAIESDLEFHHNGIHRWVDGQMAMLQTSPLDPAFWNHHTFVDYVWEAFRINSQRNGVNIETDYPANPTTMGAAELHAPDAALGFAEMTVIDGLSNTFTAEIYEYDPPPTCSVQNPDCGSKYLKCVVFRDNAHCVSRTLAEVIQWENDQTRHTTVAPTSSIPTTRPPTICPNPSPPPTHTQEDKPYQNHYCLNGKSDIRQWVYIPVKVIYRRPPEYKSYGSYPIYNGRASKTNDIYAPGAYSNVYQYLKSGQPAKYDTCAEGDKRISSIYIKSIGLNYDGIYKEYAIVDRRIAITESTAYIAVKNPAKESSLALLQATDSCGRACRPVCKVRNSNIYRPCSGAVRLTSAFPRHYGSDFGNAVMDVWDFTADKSCPQFNNANIFIAFNCDFQTDWFWPSKQPPVQQPVPPENVTRKPVDKATVVPGCDLGYGCVVNKPCAVLSEDGKDTALCPTLQYQCMNSCHMYARCWHGKLHVQQCNRGMRYDPSNLKCVPGVCDYSRSSMRLPREKRQ

> *H. cumingii* Tyr14 (complete)

MNICGTLWLLLTIWSSRSIARIMEIPLPPRMQQCFQDYSHRTSIKETVGESISWFCFDQYMWKAMRENQWMGFNITRQGYEWANGLAGVGFTQSTYRKKRQVSLRVRKEYRRMTDEERNNFHRAIQMLKEDTSVFPNKYDVLASLHQGIITTSAHGGPNFLAFHRTYLLMFENALQEKISSVTVPYWDSTMDDAMMNPTESVIWSPAFLGDGNGLVDTGPFARWQTMAGPLTRNIGQSGQLFRHEDIERIKSMVRLGQITEPNAPVNSSLEFHHSAVHIWVDGQMGTLSSASMDPVFWMHHAYVDYIWEEFRKNQYRHGVDPERDYPDVVNDTFHRADAHMGLGNLLNRDGLSNVFTSRYYTYEPSPSCSEHLPTCGSRYLKCVMIQGTPRCVSETRAVAPVVIPDIAVSRNQQFISGERQIVSNSNRFSQVSHVPTVQVGVSGSSGQSTMSLQTLPQQQQLSPQSMFSPQRVMMMNPMMQMRNAPNGDWIRQESFQGMADPRNQLMHKMQSSVPATTRMTMSANQQISMSNIKQLPINSFRTQIWTNNRGLLQTNQGNVDTMASIRAQLTNQGVTGASTVGTGNTDSSSSVRFAPLFQNISSLPDPGLCPHIPLSRGYQNTYNLNGISDINQWAYIPVKVIFQRPPTYVRYHSFPIKNGELDDAEDIYSVSSYATIKDIFLSGSPAAYSQCRTQDSGAGKVYIASKGINYMGTYKEYAVVDHRLAISIATAYVAVKSPDRGVSEVFLSAYDSCGRICQPYCKTEGSGFRPCSGAIRVTSESPKMYGSTYGEAVSNLWQFGEGNSCPIMSDDGINISFHCNYRDAWPFGHVSQNLQSKMTLDQPTQGVEGAISLGIGAQNGFGMKLNQEHGIQSKAKTEAEPADATSSYCNVGNGCVVAGQCGTCKDGAIYTCAGSCSFYTACKNGSYIMMRCPANMWFDPDDARCTPGQCVDQTNKWLYPPPNFG

> *H. cumingii* Tyr15 (complete)

MTTDPSQSIIWTSQFLGNGQGTVTTGPFARWPGGQSRLLTRGVINNRRGRLISKQNIAIVLTRCRTSDVSSPPSVGDSVIEAHHNGPHNFVGGSSGDMSFLQTATYDPVFFMHHAFVDYIWELFRQRQRTVCNINPTTDYPANVNLHEADRRMWGRNLSEYRNIDGYADFWTENWYPYEPAPSCSRQNPTGCGSQYLECDLTRNVCVSRTRSAASPAPLRRRKAVIHPLPVADDIEDLSTNIQFYPLQNTYSVDGEPDCKNWVYFPVQVVHMRPSDHTYACYPYYEGEYQKANDIYFSPVGASIYTFAKPGASKEFSKCMKYPSGAFPVYVQSNGINYYGVYTDYAIVDSRLPLTSSMIYIGVRKPPIDGVSEAILSAHDICGRVCKPQCVIPNSKPIKFRPCSGAIRLTQALPKMFKSSITDAVVYTWKLGDQMEYSVEISMAFTCLYEENWPWLTANRQ

> *H. cumingii* Tyr16 (complete)

MALAIFLFFLILIGCVTSTNAYLEFLPMPQEMTSCFNYFHQKCNLTNTVGHSLNRHCVNSYYFRSEKIKWEWTNLTDFDMHYLKFLKRRIFDNHFRAKRQSDINPSPPSGFRVRKEYRRLTDSERTAYHSVLNVMKRNGEYDTFARIHSGPNLGQFHDGPNFLGWHRIYLAYFSVMFLLLVTAAAFMSVAYALMEEMPMPPELLSCLQGFQVKTNITSTVGEEMFTFCLNKFLWKANSIKWSGYNVTEADLRFISSITTSLFHRRRTKRQTGPGNAPGTGFRVRREYRMLSDAERNAFHTALNEMKRGGQYDTFARLHAGIVVNSAHGGPNFFGWHRVYLSLFEEALRRINQNISLPYWDSTMDFDMTNPSESILWSSRFLGNGDGVVTSGPFANWNAGGAPLTRNIGGTSRLFSKQDINNILSRCRTREISERTALPQYSVEFIHGGPHNWVGGQMSGLNTAAFEPTFFLLHAFVDYIWELFRIRQRQFCNVNPSVDYPTATNLHASNRAMDGFSGYRNIDGYANYWTQLWYNYESAPTCSRSNPSCGSPYLLCDQAREVCVSRTREAIGEDVPRGFAAAESSSRSRMQEAMIDVGPRFSAPPSDGRSVDVQGGILGGASTVQAELLGASMGPRFNPPPSDGRTADAGFAAFSGINSDKVHEAAAETIVPQPSIIPGGNLIRPIQNTFLLNGVADTSKWSYVPVQIIHSRPNGEIYSSFPINGGIVNLQQDVFSPEQYPQLKAFINPGHPSGYEHCKSDPSGAAQIYVQSDGMNYNGRYADYAVIDARLPVGSGMVYMGIKQPSNAMSDVIFSAHDRCGRMCQPQCLVNGTRPPIYKPCSGAVRFTPSTPKMYGNTFGDAVMSVWNMINGDPQNYGGSVPLVMACNYKDSWPWDHCNQGSGQ

> *H. cumingii* Tyr17 (complete)

MSFCSFLSVVMISLLPYVRATVEQTNVPDELTWNFYVAQHKTSRLNTPSNAIQSWCTNVYKWQHDSIVHGGRNASTGTRQLVNYFSDQVHLSVRRTGHAKRQVAPSGPKRRRKEIRMLSEKELDLYFRAVRAAKANTTTAPNVYEALAEFHTGITSISAHGGCNFFGWHRVFLLMYENMLRDQGPEFAEVTIPYWDSRLEARMDQPTSTVLFTDRFLGTGSGEATGGILGSGWTTSAGPLVRNIGTDGPPMTDEAIVNVTRMTRMREICGADSSIESDLEFHHNGIHRWVDGQMAMLQTSALDPAFWSHHTFIDFVWEAFRINSQRNGVNIETDYPANPTTMGAAELHAPDAALGFAEMTVIDGLSNTFTSQIYEYDPPPTCTVQNPDCGSKYLKCVVFRDNAHCVSRTLAEVIQWENDQTRFTTVAPTAPIVPTLPPSGCSNPSPPPTHTQEDKPYQNHYCLNGKSDIRQWVYIPVKIIYRRPPEYKSYGSYPIYNGKSSKTNDIYAPSAYSHVYQYLKSGEPAKYEKCVEGDKRISSVYIKSVGLNYDGIYKEYAIVDRRIAITVSTAYIAVKNPATESSLALLQATDSCGRVCRPVCKVPNSNIYRPCTGAVRLTSAFPRHYGTDFGNAVMDIWDFTAEKSCPQFNTENIFIAFYCDFQTDWFWPTKEPPTQQPVPPGHVIHKPGKAYGTGMGQNPQVGAAPITDNAAAVPGCDLGYGCIVSKPCAVLSQEGKDTGLCSTLQYECTNSCHMYAKCWYGKLYVQQCNRGMRYDPSSLKCVPGVCDYSHSSMGLPRG

> *H. cumingii* Tyr18 (complete)

MIWFISLPVVMISLVPYVKATVEQTDVPEELAWNFYVAQKKTSTSNTPANAIQSWCTNVYKWQHDNIVHGGRNVTTGTRQLVNYFSDQVHLSVRRTGLVKRQVAPSGPKRRRKEIRMLSEKELDLYFRAVRAAKANTTTAPNVYEALAEFHTGITSISAHGGCNFFGWHRVYLLMYENMLRDQGPEFAEVTIPYWDSRLEARMDQPTSTVLFTDRLLGTGSGEASGGILGSGWSTSAGPLIRNIGADGPPMTDEAIVNVTRMTRMREICGADSSIESDLEFHHNGIHRWVDGQMAMLQTSALDPAFWSHHTFIDFVWEAFRINSQRNGVNIETDYPANPTTMGAAELHAPDAALGFAEMTVIDGLSNTFTSQIYEYDPPPTCTVQNPDCGSKYLKCVVFRDNAHCVSRTLAEVIQWENDQTRFTTVAPTAPIVPTLPPSGCSNPSPPPTHTQEDKPYQNHYCLNGKSDIRQWVYIPVKVIYRRPPEYKSYGSYPIYNGKSSKTNDIYAPSAYSHVYQYLKSGEPAKYEKCVEGDKRISSVYIKSVGLNYDGIYKEYAIVDRRIAITVSTAYIAVKNPAMESTLALIQATDSCGRVCRPVCKVPNSNIYRPCTGAVRLTSAFPRHYGTDFGNAVMDIWDFTAEKSCPQFNTENIFIAFYCDFQTDWFWPTKEPPTQQPVPPGHVIHKPGKAYGTGMGQNPQVGAAPITDNAAAVPGCDLGYGCIVSKPCAVLSQEGKDTGLCSTLQYECTNSCHMYAKCWYGKLYVQQCNRGMRYDPSSLKCVPGVCDYSHSSMGLPRG

> *H. cumingii* Tyr19 (complete)

MSFCSFLSVVMISLLPYVRATVEQTNVPDELTWNFYVAQHKTSRLNTPSNAIQSWCTNVYKWQHDSIVHGGRNASTGTRELVHYLSDQVLQSVHHIGRVKRQVVSSGPKRRRKEIRMLTERELDLYFRAVRAAKANTTTAPNVYEALAEFHTGITSISAHGGCNFFGWHRVYLLMYENMLRAQGPEFAEVTIPYWDSRLEARMEQPTSTVLFTNRFLGTGSGEVSGGILGSGWQTSAGPLIRNIGTDGPPMTDEAIVNVTRMTRMSEICGADSAIESDLEFHHNGIHRWVDGQMAMLQTSPLDPAFWNHHTFVDYVWEAFRINSRRNGVNIETDYPANPTTMGAAELHAPDAALGFAEMTVIDGLSNTFTAEIYEYDPPPTCSVQNPDCGSKYLKCVIFSNNAHCVSRTLAEVLEWENNQVTRTTVAPVTSPPPTACPHPPTTQLYTEHDKPYQNHYCLNGKSDIRQWVYIPVKIIYRRPPEYTSYGSYPIYNGKISKFYDIYAPNVYSNVYQHLKNGQPAKYEKCSEGDKRISSIYIKSVGLSYDGTYKEYAIVDRRIAITVSTAYIAVKNPAMESTLALIQATDSCGRVCRPVCKAPNSNIYRPCSGAVRLTSAIPRHYGTDFGNAVMDVWDFTTDKTCPQVKYENILIAFNCDFQTDWFWPTKQPPAHRPLPSGHKIHKPIHGPKLVKG

> *H. cumingii* Tyr20 (complete)

MTTDPSQSIIWTSQFLGNGQGTVTTGPFARWPGGQSRLLTRGVINNRRGRLISKQNIAIVLTRCRTSDVSSPPSVGDSVIEAHHNGPHNFVGGSSGDMSFLQTATYDPVFFMHHAFVDYIWELFRQRQRTVCNINPTTDYPANVNLHEADRRMWGRNLSEYRNIDGYADFWTENWYPYEPAPSCSRQNPTGCGSQYLECDLTRNVCVSRTRSAASPAPLRRRKAVIHPLPVADDIEDLSTNIQFYPLQNTYSVDGEPDCKNWVYFPVQVVHMRPSDHTYACYPYYEGEYQKANDIYFSPVGASIYTFAKPGASKEFSKCMKYPSGAFPVYVQSNGINYYGVYTDYAIVDSRLPLTSSMIYIGVRKPPIDGVSEAILSAHDICGRVCKPQCVIPNSKPIKFRPCSETIRLTQALPKMFKSSITDAVVYTWKLGDQMEYSVEISMAFTCLYEENWPWLTANRQ

> *H. cumingii* Tyr21 (complete)

MTTDPSQSIIWTSQFLGNGQGTVTTGPFARWPGGQSRLLTRGVINNRRGRLISKQNIAIVLTRCRTSDVSSPPSVGDSVIEAHHNGPHNFVGGSSGDMSFLQTATYDPVFFMHHAFVDYIWELFRQRQRTVCNINPTTDYPANVNLHEADRRMWGRNLSEYRNIDGYADFWTENWYPYEPAPSCSRQNPTGCGSQYLECDLTRNVCVSRTRSAASPAPLRRRKAVIHPLPVADDIEDLSTNIQFYPLQNTYSVDGEPDCKNWVYFPVQVVHMRPSDHTYACYPYYEGEYQKANDIYFSPVGASIYTFLS

> *H. cumingii* Tyr22 (complete)

MIWFISLPVVMISLVPYVKATVEQTDVPEELAWNFYVAQKKTSTSNTPANAIQSWCTNVYKWQHDNIVHGGRNVTTGTRQLVNYFSDQVHLSVRRTGLVKRQVAPSGPKRRRKEIRMLSEKELDLYFRAVRAAKANTTTAPNVYEALAEFHTGITSISAHGGCNFFGWHRVYLLMYENMLRDQGPEFAEVTIPYWDSRLEARMDQPTSTVLFTDRLLGTGSGEASGGILGSGWSTSAGPLIRNIGADGPPMTDEAIVNVTRMTRMREICGADSSIESDLEFHHNGIHRWVDGQMAMLQTSALDPAFWSHHTFIDFVWEAFRKNSQRNGVNIETDYPENPTTMGAAHLHAPDAALGFAEMTVIDGLSNTFTAEIYEYDPPPTCSVQNPDCGSKYLKCVVFRDNAHCVSRTLAEVIQWENDQTRHTTVAPTSSIPTTRPPTICPNPSPPPTHTQEDKPYQNHYCLNGKSDIRQWVYIPVKVIYRRPPEYKSYGSYPIYNGRASKTNDIYAPGAYSNVYQYLKSGQPAKYDTCAEGDKRISSIYIKSIGLNYDGIYKEYAIVDRRIAITESTAYIAVKNPAKESSLALLQATDSCGRACRPVCKVRNSNIYRPCSGAVRLTSAFPRHYGSDFGNAVMDVWDFTADKSCPQFNNANIFIAFNCDFQTDWFWPSKQPPVQQPVPPENVTRKPVDKATVVPGCDLGYGCVVNKPCAVLSEDGKDTALCPTLQYQCMNSCHMYARCWHGKLHVQQCNRGMRYDPSNLKCVPGVCDYSRSSMRLPREKRQ

> *Pinctada* *maxima* TyrA2 (complete)

MSKMPSLLQILYLILAVFPLCRLQQRSVNNLNPRFIMWMNSLFYIPNDRNLRVRKEYRMLSDAERRDYNRAIILLKNDRTVSPNKYDALASLHHLNSANGAHGGPGFLGWHRVYLVLFENALREKVPNVTIPYWDSTLDSDLPDPRRSIIWSPLFLGNGNGPVVNGPFRRWSTPYGPLRRDIGADRRLMNRQDIQNVFSRRWLWEITNPSARDEYNIELLHNHVHVWVGEQMSRIESSSYDPAFFAHHAFIDCLWEEFRQRQRQQGINPARDYPRIVGDQNHQPLVSMGLGRLLVIDGINDFFTRQIFRYERRPVCVRGSNTCGSPYLRCNWSTQTCLPLIMSNRGTQTRRVVQNRRQPWWRRFVNQRNTFFG*

>*P. maxima* TyrA3 (complete)

MKGLLWRFFLLVGLICVVYPDIIEIRVARELEECFEQRRFDTNQTDPRYDNIHGYCIQNFRWHLQEHYWKNITMETSNWIEELLRISNRKVRKKRQSLPVRKEYRRLTDQERADYHRAINMLKRDTTVKPNRYDALGLLHQRRGDDVHHGAGFLGFHRVLLVVYENALRQKVPTVTLPYWDSRLDQPLRDPTRSIIWSPQFLGTMRGRVINGPFAFWQTPAGPLVRNGGQEGELFTYNHIRAVMTRSHLEEISEPHAPPPFDFEIRHGDVHQMVGGIMAPAETAGYDPVFFLHHCFVDYLWEVFRRSQKEKGVDPTKDYPRRYGPAAHAPKEQMGLGRLLNEHGLSDMFTSRLYTYEPSPTCSYRRPTCGSNYLTCEFGFGRPQCVTLEMLSTTPTQSSRSNIPLQWRQFVPQLQTGVPRSPVPNQFRSALARAQARQQGNTLPNRAPNPNQFGTPRRFGRTKRQADAHAIDGGRLTQMGQNILSQFGGNQLSEFIRNKTKPLPEKAIPKLYKQRHHKRIFSK

> *P. maxima* TyrB1.1 (complete)

MNTMTLLGKVFLLQFLIGVGFCMLMQDPKRNDTKGTYAACFRSQPQGNEPASPDCLKAFMAYAEDMKNIFHFTKEQINYLWSLERETQSLLHNHRRRKRQAVYLPVRKECRLLSELERQNLFYTVRSLKMDTSNPNEYDTLANLHRGAVQPHAHDGSNFLGWHRVYLMYYERALRRIRGDVTLCFWDTTMEFNLGMDNWEYTAVFSSDFFGNRRGQVITGPFRDWPLPPGLTESDYLYRNMTRGRGMPFDSRAASSIFYNPNTIIHSTITWEGFGFDTITNSQGQTRNITIEGEHNNVHNWVGGAMGFLDPAPQDPIFFFHHCYIDYVWERFREKMRRYFRDPTTDYPGHGNETLHDANYPMIGFEWYRNIDGYSDYFTQNVYRYESPTCQACYYSPYTVCGQGNQCIARMNYPGTEIEEGPQVPNGPVAAFSVAGGTMMMSASNGRGFIATSNSE

>*P. maxima* TyrB1.2 (complete)

MTLLGKVFLLQFLIGVGFCMLMQDPKRNDTKSTYAACFRSQPQGNEPASPDCLKAFMAYAEDMKNIFNFTKEQINYLWSLERETQSLLHNHRRRKRQAVYLPVRKECRLLSEMERQNLFYTVRSLKMNTSNPNEYDTLANLHRGDVQPHAHQGSNFLGWHRVYLMYYERALRRIRGDVTLCFWDTTMEFNLGMDNWEYTAVFSSDLFGNRRGQVITGPFRDWPLPPGLTESDYLYRNMTRGKGIPFDSRAASSIFYNPNTRIHSTITWEGFGFDTITNSQGQTRNITIEGEHNNVHNWVGGAMEIIKPAPQDPIFFFHHCYIDYVWERFREKMRRYFRDPTTDYPGHGNETLHDANYPMIGFEWYKNIDGYSDYFTQNVYRYESPTCQACDYSPYTVCGQGNQCIARMNYPGTEIEEGPQVPNGTVAAASAAGGTMMKSASNGRGFIATSNSE*

>*P. maxima* TyrB3.2 (partial)

MRYLKFRTSAYDFFAGLHRSLRSFRNAHIGSNFLGWHRVYLWYFERILIRVGGVPLCYWDSTLDFRIEGSGQRNTTMFTSEVVGNGIGMVINGPFRNWPIPDRNVSLRREIASFASLMRPQVVDLIMTSNLIRNHSQISNGAGSVGMIDPDQGTRTSLESEHDNTHVWVGGVMSDATIAPQDPVFWLHHTYIDYVWEKFREKLFTLGINPANDYPGHGGDPHAANTQMVHFYNFSNWWTNENGYTNLFTQFVYTYDEHPTCGNGCGGRGDTNLLYCPTGGTGDQRCVATVVVWSA

>*P. maxima* TyrB3.1 (complete)

MRYLKFRTSAYDFFAGLHRSLRSFRNAHIGSNFLGWHRVYLWYFERILIRVGGVPLCYWDSTLDFRIEGSGQRNTTMFTSEVVGNGIGMVINGPFRNWPIPDRNVSLRREIASFASLMRPQVVDLIMTSNLIRNHSQISNGAGSVGMIDPDQGTRTSLESEHDNTHVWVGGVMSDATIAPQDPVFWLHHTYIDYVWEKFREKIVHFRHKPSQ

>*P. maxima* TyrB4 (partial)

MKDGPESMYDTFARVHQNQESLDNAHGGSNFLGWHRLYVLFFENALRRIAPGLVLCYWDPTLDYMMKSTLQIHSVTFSDRLFGNGYGTVINGPFKNWQLFEPYNYRLRRNIGQEGSLTRPEVIDIITLNPKIIRSTQISSGLGAIGFKDPDTGRRHSLEQCHDNTHVYVGEVFSSLPITAQDPIFWFFHAYVDYVWELFR

>*P. maxima* TyrB5 (complete)

MDRYRGLKNLLNYTEDQMNYLFSLERAMMRKHHINNKRHKRQAMMRPRQECRTLPDDARNNLFNTIVDLKASSNGMSQYDTIAGLHSLQAFPNAHQGANFLGWHRIYLNMFETALQESRSDVVLCYWDSTLDFLMPENTQLNTVTFSAELFGNGRGSVINGPFRNWRLPGGRTLQRFIAGPGSSLTRPGVVDLIATDPRINSHSQIVVGGQGFPDPDTGRPGHSLESEHDNTHVWVGGVMQNVVLSPQDPVFWFHHTYIDYVWELFRQKIGPDAREQYPADASGQHAPDAPMVGFDMVLNRDGYSDEHSKMYAIHPRCSDNCGNSRFLECLDNGPMADPNRRCVSRAVNSDMVPAAAMSAPAAAGFGFSAMSPMGAFGPAGFGPSSVGRMAPLGRAARVSLQATDTVAIRAAMSEPPLELEGPSFQSSFDDPRI

>*P. margaritifera* TyrB1 (complete)

MNTMALFGKVILLQFLIGVGFCMLMQDPKRNDTKSTYATCFRSQPQGNEPASPDCVKAFMAYAEDMKNIFHFTKEQINYLWSLERETQSLFHNHRRRKRQAVYLPVRKECRLLSELERQNLFYTIRSLKMDTSNPNEYDTLANLHRGAVQPHAHDGSNFLGWHRVYLMYYERALRRIRGDVTLCFWDTTMDFNLGMDNWEYTAVFSSDFFGNRRGQVITGPFRDWPLPPGLTESDYLYRNMTRGRGMPFDSRAASSIFYNPNTIIHSTVTWEGFGFDTITNSQGQTRNITIEGEHNNVHNWVGGAMGFLDPAPQDPVFFFHHCYIDYVWERFREKMRRYFRDPTTDYPGHGNETLHDANYPMIGFEWFRNIDGYSDYFIQNVYRYESPTCQACYYSPYTVCGQGNQCIARMNYPGTEIEEGPQVPNSPVVAFSVAGGTMLMSAFNGRGFIATSNSE*

>*P. margaritifera* TyrB2 (complete)

MMMPYGLTLILLLFTLSDATLLGIKYDPIPRCAREIMADHTSPNYNSTFNNDCAEFISNSFRELKKLLNFTDDQINFIKSLDREAMSLLYGSERTKRQTGLHVRRECRTLSQNDWGRLAQAIRRLKFDPGNEYDTMAHTHTLPAVIDNSHDGSNILGWHRLFLFLFEIALRRKVGGVVLCYWDSSLDYLLRGRGQVQSAAFSHELFGNARGQVTTGPFANFPTPWGPLRRNFGGEGGSLVRPHIVDMIERDPNIRRDGQLVDGDGATGFTDPLSGERTSLEAEHNNAHVAVGALMAIIPNAAYDPLFFFHHCYIDYVWELFRRKQMRLGIDPTRDYGGHGGPAHARNAPMRGLIPGWRNIHGYSNFFSRRYRYAYHPVCGNGCSGSERFLYCPRGRRFRRCIPRTMEGRARPPQRIVGRSRGASDITFSTNYDDSTIAH

>*P. margaritifera* TyrB4.1 (complete)

MQLLTVIVLSPLLVASVLTINVRLRNLMETQDFKTCFGLNTTLYNLYGLDYRVPECTEIFMSTVKFSNLTKKDVHLMDSLGREVLSELQDSSRNKRYIKPLRIRRECRTLSDKARNRIFDAIVKMKEGSENMYDVFARIHLSPETLDNAHGGSNFPGWHRLYVLFFENALRRIAPGVVLCYWDPTLDFIMKSTLQIHSVTFSDRLFGNGYGTVINGPFKNWQLFEPYNFRLRRNIGQEGSLTRPKVVDIISLNPKIIRSTQILTGSGAIGFKDPDTGRRHSLEQCHDNTHVYIGGVFRSLPVTAQDPIFWFFHAYIDFVWELFRTKQKKHGIDPSKDYPEHGGEDQRALHRMVPFFAFKNIDGYGNMFTEKIYRYAPSPVCGNRCGGASKELLYCPRGGHRRSRCVSRARNVDFVPNRTLKEIHNAYLEKFVRGNKVSKGILCGISLEVAKMDGKVNLPFPLFKAPFDDPRVFVGKGYEENWRKYGG

>*P. margaritifera* TyrB4.2 (complete)

MYDVFARIHLSPETLDNAHGGSNFPGWHRLYVLFFENALRRIAPGVVLCYWDPTLDFIMKSTLQIHSVTFSDRLFGNGYGTVINGPFKNWQLFEPYNFRLRRNIGQEGSLTRPKVVDIISLNPKIIRSTQILTGSGAIGFKDPDTGRRHSLEQCHDNTHVYIGGVFRSLPVTAQDPIFWFFHAYIDFVWELFRTKQKKAWDKSL

>*P. fucata* TyrA1 (partial)

IEDLIQRKLRNILQHTFTSMENHKSYSFSMEGAWNNLFARTECCGIESIKDFHAITSNQILGPVLANIPVYCCTTNPFSELYHGQDTKCTVNLEKDLRFNTSVQPNLYDAICNLHPNVRAPNAHYGPAFLGYHRVLLWFFEKQLQRKVPGVYLPFWDSTKDSLMANPTASVIFSQAFAGTGNGVVNNGPFANFRHPAVGVLVRNLEAGGDLMTRRDIANIVRQTRTADMMTPYASSENNLELCHAKVHAWIGGTLDNLNYSPADPLFWMHHCYVDYIWELQRQNERRGGRDPDTDYPSMDDGHNADGIMYPFTNLRNKDGFNRGWIDTYYGYEDAPVRCRRDDSCRSTYYKCTRGLCCSRTIGEVYGVGQQQTFGRKKRSAPTKKSSTYSGKASSLVKKAPVVTKPVTDIQTHLKSYFPYLAKLGDSAGTFYKFKHGQSPAIKPGPEKVQDLSFEDLDLHTTGLYHSFQNSFTLDGVEDTKNWVYLPVIVYYRRPAEVKYDAHPIRNGKVMANMDVYSTTRADKYHLGKPVKTATSSTCKHIGSGAFKVYVKAYGLSYDGVFTDYAVVDERQPLSFALTQVGVQNPRMKNTTVYVTAYDSCNRQCKARCLIPGSKPARYKPCSGTIRVTPDAPFMYSSDIADAHLHTYDYNTIPPCLSYDKIFLVFYCDQKDSWPWQQSMGPAHVSSARTAGGGYTPPSMPRYAPQRMPVHVQAHRFASQHNFGFRPPQNFAAFHGKFLKY

> *P. fucata* TyrA2.1 (complete)

MSKMPSLLHILLLIIAICPLCRLQQRSVNNLNHRFVTWMNSLFYIPNDRNLRVRKEYRMLSDTERRDYNRAILLLKNDRTVSPNKYDALASLHHLNSANGAHGGPGFLGWHRVYLVLFENALREKVPNITIPYWDSTMDSDLPDPRRSIIWSPLFLGNGNGPVMNGPFRRWSTPYGPLRRDIGADRRLMTRQDVQNVFSRRWLWEITNPSARDEYNIELLHNHVHVWVGEQMSRIESSSYDPAFFAHHAFIDCLWEEFRQRQRQQGINPARDYPRIVGDQNHQPLVSMGLGRLLVIDGINDFFTRQILLDMNVVQLCKGVHTCGSPLPEM*

> *P. fucata* TyrA3.2 (complete)

MIPILHAIAIVICTCATLSSGLIKYRELPDRLAVCYNQHYKACSTQTKVGLSILHRCMSEYHWKSGPQRDYPGRTPTPAATRYVNTLTRDFQPGGATRFRGKRQSRSRVRKEYRMLTNDERGRFHRALQALKQVSSGEDQSRFDMIASFHTTAETNAHGGCNFPGWHRYYLLLFERALQSVDPSVNLPYWDSTLDQYMDESAQSLIWSEDFLGNGDGAVTSGPFANWETSEGPLVRNIGVPDTQLYTHEQIFNTTRSTRMSQICNGQTDSEVDPLVNTGLEWHHGDVHVWMGGQMSMLTTSSYDPVFFMHHAFVDLIWEEFRQNSRRAGVDINRDYPTANYGEHDFHGPDAPLGLTTNLTVAEAIDDENIPPQVRYRYERRPSCGRNRDCGSDKLRCVQRGRNYICISKTLVEYQTDLQIEQATNGGNVNPNNGNTVIIRRPDPSPNNPNRGNRGPTFINPRRNRMINRIRGSTRWTFRDRLMRFGIRNTVAVIDQCPVPEHEHKPIQNTFCANGNSDITRWVYIPVRVLAQRAPGFQKVRIVPDS

> *Crassostrea gigas* TyrA1 (complete)

MRKGQLNFIVCFLATVVLPTSFGLIEEIQTPRDILECLIYKSQNSTIGEVSGRTIQDFCIRKYTLDTQSGKENFAKNISTEGVQYLKSLFRQLESEVHDQKRGKRQAGTWRVRREIRTLSDAERNNVFQCLRRLKGDYSIDPRMSTYDLIASLHSGQAARMMHNGPAFLPRHMVYLLVMETACRVPMPYWDMTTDSEMMDPTTSIVWSDLFFGPGNGPVLTGPFGRFRTPTGTPIIRNIGSGGASLARKAGIRALLSRRRTFEISEPQPAQSIFSIEVHHNGVHNYIDGYMSGLNTASWDPVFWFIHSFFQLLWVAFRNGQRANGINPERDYPRGVRVPAGHEFYQRMNFMPFMRRITNLEGLSNRYDRIVQYAPMPRCPACGGSPFLVCLRGVCVSRSSRRAPVFFRGKRSAGTSGDQLADENVNPNTTNLIQSNEAALSTLNKPYQNTFMIDGKIDEDAWAYIPIRVLYERPKGFNFHTTSPGATKRDMYDPENFKNEAKRIGLHNEVQYKQQCTPSGSGAAKVFVQSNGLNYAGKYKDYAIVDERQPVTSAITYVGFKKPSTSDSEVILTAYDTCGRICRPACPVYGHHRESYRACSGSFRISSKSPLTFSQNYGSAVSSTWNVREMEPGCKSSQSLPITFVCDHQNSWPWESKL

>*C. gigas* TyrA1.1 (complete)

MEPSTSDTFADNITYFSHNSGSFVYDTFGINGGYEPYGTDSICNRHSKHDYEIPEQVCQAKMFFFTLMLIVINARFEIAYSKMYEEPLPQEFKDCLNMILPNSDLKRDPAYIIDYACANKFLTSTPNKRWAPEKDEEEFTLITNKINKLDIHTSSENVRYEKRSIQNHLDNRHKKRSAVNPTIRRKEYRRMSPAERTDFHRALQLLKDDAERTKFNLYDILCNFHHASRAPHAHFGPAFLSWHRVSLYIFEQALRSKVPGVSLPFWDSRLEAALENPSATSLFTDELMGPGNGVVKTGHFANWSHPFAGDLVRNVGNLGEPIQRRDIERLLKARYTKEFMFPSAYPHMNLELIHGKVHMWVSGTMNNLNYSPADPIFWMHHCFIDYVWEKIRQRQKERGVDPSYDYPMNGGIGHRPYDTMKPFDLKNRDGYLIDWSKVYQYETSPVDSKCRSDSDCGPEYYVCANGNCRAKTAEEVLFDRRSRRRRSIADLGTSDLLSKDHYNDLQTSTDAQTTPVFHSMQNTFMIDGREDSKSWVDLPLIVYSKRPDHLVFSAHPYRRGEANFTADIFQPTLKEENVLPRSGNPAIKQKCKHMGSGVSKVYIRSIGVNYEGDYTSEAIIDERQALTITLIQIAVQNPYKKYTKSYISAYDQCGRMCSPYCLTGRDGTYKKCSGNIGVDSRIPRMYNTDAAGTILQMFDFKSFPPKLDQSNIFLIFYCGQEEKWPWEINKEEK

>*C. gigas* TyrA1.2 (complete)

MTSYYDDVIIAMSHNVTCTLTSFDNEVDPDLSRAALATCALYASLQRPVTTASSCPATTCPNGWTRYQTSCYLVVTYELETWSGAQAKCVAENSGLVEIETEAENNFLKDVAAKTFLNGQFWTGGNDIDVEGQWRWVTSGNPFTFTDWGPGEPNDTGGNEDCMLLLSNTGYTWNDLPCSTNSLYICEKPRSIDKLALMVKPTKTISSCVCVPCCKMVEKISTRMGTGGGWIQYLLVYLYITAPCHALVYEEKMPHLLRNCLERTSHKKGNVTRDTAESIDYMCTKEYLFKTPEERWHPDLDQVINTKTLKKFASLFKELDIGETRSHKIRYRYSSIKRHFVRVRRATPIVRKEIRMLSEEERQKFLKALVAMKADTSDPQRQPNVYDWFCNLHPNKVAPNAHYGPAFFGFHRIYLYLQQQLRTYEPDTFLPFWDSTYESLLGTPTSSVLFTEDFLGGGTGTVTDGPFKNWKHDKVGVLIRNTANSGQLFQRETIDKIMMKMFMSDISNPDAEFDVNLEAKHGQVHAWIGGAMDNLDYSPADPIFYMHHCFVDAIWERFRDNQVKRGMDPTSYPDVTEGHAANKPMKPFYLEVKNGKKIYLKNKDGYALKWSQLVRYVYPKRGCKRNSDCQSDVMVCREEQCMTMTAHEYEASKAAPPPPPPPPPAPPKRRHSSSVYKAPINKVPITAAFITTTPAPTRGVDDLPGWDNWFMWRKKRSVSYVHTRYPYPYHYIRRHLYYLTRRYRKKSRQQRREKIGEVGIKYKYSDEKTSPIYHSLQNTFTIDGVEDIDNWVYIPIIVYYRRPNEVHYDAHPISHGHPIMNKDVFNQYHDAKYITHKVGNPAKNANCYHIGSGATKVYVKATGMNYNGFYTDYALVDERQPLSFGLTEVGVKKPVGKTPSKVYISAHDPCGRSCKARCLVAGSNPPRYRPCSGTIGVTSKGPLMYSSNIGDAHLNTYDFSTLPPTLSSKKIFLVFYCDQSEVWPWDA

>*C. gigas* TyrA2 (complete)

MSITPSTKITKSIAHGSGVPGPKMSRERTAMFAVAFALVALPTISGLITKIPTPKELYECYLYKSLNASISETPAKVIQEFCISKYIVQHMDETIYTHNITKEGVNYLNAMSRELNQEITAMEKLQKKPRHKRQAFGTWRIRKEIRTLTRQEFQALVNCFNRLKNDYSIDSSMSTYDLIGSMHTGRAARSMHNGPGFFPRHMLMMLIMETACHSPMHYWDMTMDSDMIVPTDSIMFSEEFYGNGDGIVRTGPFAHWRTPIGTPIIRNIGSGGESLAGKQGIRAMLSRRRLAEISEPQVGHAAYSVEVHHNGVHNWIDGHMARLNTAWFDPIFYGIHSFFTLIWIAFKGLQRNRGIDPQRDYPLGPNVPSGHEFFQRADFRPFLRQISNLDALADTYDRMVTYMPMPRCPSCDNSPYLVCQRQVCVSRARPRMRQNMFMFGRKKRSANVNPTPSVYGTQKPLSDSDSLADDNVDNKTINILQSSETALSTLDQPYQNTFLIDGKSDLNQWVWLNIKILFERPKGFNFHTNLPGQNNTVDMYDTSNFERIAEKIGIHNQITYKKQCSSSGSGAAKVFVQSDGLNYAGRYKDYAIVDERQPIYSSVSYIAVKKPTTAKTTKVLLTAFDTCGRSCRAACPVPGTNGNSYEPCKGAFEITSEYPLMYSTSYGGAVSAAFSVHLESKGPMYRVNNHPITFVCDHQNKWPWE

> *C. gigas* TyrA2.1 (complete)

MFATSDPRFEFPLTEADVTWLNSLFSLPEEGETRERKEYRLLTEEEREYFHRAVNMLKNDTTVSLNKYDLLANIHSRSSSNTAAHGGPGFLGWHRVFLLLFENALRQMIPTATLPYWDCTLDQPLSHPSESVIWSDLFLGNGDGEVNTGPFRGWNTQFGFGLLHRQVSSLRHLMSVHDLRNILEEDFLGNISYPDTKSSKNLEQLHNNVHVWVGGLMRKIEIGAFDPVFYVLHTFIDKVWEDFRVHQRSKEIDPTKDYPEFYGRRNHASFAPMGLGNLVVIDGISDVWNKNVVYRQPSCGNSGQNECGSKFLRCDHSTWTCVSKTKDEVTKSINKDKHKDTTLSRTLNSGLKELDNLYIPSLLNSGISHSVYSDFLYPLKDRQSTAMYSENVSQSSALQKAKEIYMSHLN

> *C. gigas* TyrA2.2 (complete)

MTRKKPDIRLSKPVVVHRETTELSKMCPVLKVSGRMFPVEIEWMKTSYGSEIADEYEIKAIEKAAEIHGREPPGDILVFLTSQLEIEQCAKKLEILLRGMKDYWILPLHGKLQTDEQKLVFKDSPIGRRKIVLATNVAETSVTIPGIKYVVDTGAVKELSYDPRKKEYVYKDFKFQTMPSLMSVLHVLVLFLAIFPISRLQIRTQTNANSRFVQWMNGLFYLPRGNELRVRKEYRLLSDEERRSYHQAILLLKNDRTVLPNKFDAIASLHHLNTASGAHGGPGFLGWHRIYLTLFENALREKVPNVTIPYWDNTLDAELPDPRRSIMWSPLFFGNGNGAVVTGPFRRFTTPYGPLRRDIGADRRLMSKTDLENVFSRRWMWEISNPSAEDRYNLELLHNHVHVWIGEQMSRIESSSYDPAFFSHHAFVDCLWEEFRQRQRQHGINPGRDYPRIVGDQGHQPLVSMGLGRLLVVDGINDIFTQRIYSCQRRPECVPGTNHCGSPYIRCNWSTRTCLPLIMSNRPNNPVAQGPAQQQVPWWARLMQRNGLFG

>*C. gigas* TyrA3 (complete)

MYNKKYYLFSAALAVCLPFIFGIVQEKPTPTELQECFFLKSLNTSVAEVPGKLIEDFCLRKYSLSQFEGKTQKNISVEGVRYLESLFRQIDAETQLTRKKRQATATWRVRSEIRTLSAAQRNRIFGCLNRLKRDYTIDRNTNTYDLIGSLHSGQSAQLMHNGPGFLGRHALYVLAMETACRTPIPYWDFMMDGALNNPTSSAIWSNTFFGNGNGAVRTGFCGNWVTPQNTPIIRNVGAGGVRLPRRDALRAILSRTTTREITEPLPTMSMFSIEVHHNAVHNYVDGHFSALDTSTFDPVFWFLHSMFHYMWYMFKNNQRARGVDPQRDYPRGPNVPQGHEYFQRVNFMPFVRPMTNLETFANRYDNIVRYTSLPRCPDCGGSQYLVCIQGECIARSVRTTRNPARISRRPGVLPVRQFVRGKRSTETDNFSLTQNHAELLSVLDRSYTNTLVINGHYTPKDWVYLNVRVIYERPKGNMFNSTGSVNGRDMYDPSSFHDGGKEIGIKNQVFYKQRCVPSGSGATKVFVQSDGLNYHGKYKEYAIIDERQAVSSAILQVAVRKPSDENTETYLSAYDSCGRVCRPICPMKEGSLLAYKACSGSFKITNEYPLMYSNSYEGAISSLWHLSLDGKGPLFKSTFSPITFVCDHQNVWPWSQK

> *C. gigas* TyrA3.1 (complete)

MKISWIYSLLTISTVFLSQMKKHLCIVFTFIVLVGAYIEEIDLPESFTECLEKQRIKNLGAASGEAMSKWCMNSHRMKLTGDKFKYSNVSEDTVSWINELLRMSNVDMKLDLSSTNKLSHSVKKRQASLDTPSLYPNVQFPPQPIIDSDSSDSSNIQNSQGPEYMSETLQSFSNQHTGSGQAQRFQPGQARPFPIIGGENQAPAAQKPGQQQTVFQQPSQQINNFVAAQQQPTFAQGQQQNFQSMNAFQPQLQPQPTVMSPNMNTAQPVSGIPVVQRPQLRIRKEYRTMTEQERANFHRAILLLKQDTTIRPNRYDALGLVHFRMVDNIHHGGAFLAWHRLFITIFENALRQKVPDVTLPYWDSTMDEAMIDPTQSVTWSPQFLGNGDGLVTTGPFAFWQTPNGPLIRNVGQDGQLLSRQAIMRVLSRTRMAEITEPGAPDQYNIENYHGDAHTWIGGQMEPMETSAFDPVFYLHHAFVDYVWEIFRQQQRAMGIDPTQDYPQNFGPQSHAPFTPTGFGNLPNVFGISDMFTTQVYTYQPHPTCSFQNSNCGSPFLTCDISAGVPQCIPIGAGPPAPPRAAAMRGVPGVMGAGGPGPLLGGMPRLPFGRKKRATTANKASDKSTTADSYHQVMVKQLQCSNAWVSFSSQNTFEINNEGDTRNWVFIPVRVVNKRSPEHRKFKAYAIINGKPSVKADIYDPEQYEEIKPYFSEELMPSSAKCDAVSGDRTIGRIHIRSDGLNYQGNYDEYVIVDLRQAFTESTTYIGLRRPGKNDTEVLLTAYDSCGRKCKAFCRSRNAKSHDYHPCSGALRVNSNSPWEYGKNYGDAVRMSWDLNDLYTIPKLQDQSVFIQFFCDHR

>*C. gigas* TyrA4 (complete)

MYSLVALCGILTFHFGYSMIEEMTTPGDIEDCFDGYYRKTSLQRSIGSKIYWKCMQRAACNRAMLNLGSNMTLEERHYIESLLPPPEVFYGNGAKGFHPNSEHRWRKEYRMMTEKERQAYHFAVNKLKRLRLGTSNRYDVIAALHEGAIVNAAHEGPNFMGWHRIYLIVYENALRQIVPGVTIPYFAGDLDEPLRDSTQSVLFCERFFGNGNGVVTSGPYANWSTPSGPLVRNYGDDGELWTREGLQRILNKTRNAEIIAPNAEEEDNLEDQHGAIHNWIGGGNGQIGELQTSSQDPAFFSLHAYVDYIWEEFRKRQASLGINPAKDYPVDYGPEEHHPLRLAGFATLRNIDGYSHGLASLVSYKPKPTCSKDRPFCGSPFLRCDKKSNPPRCVSKTIASFYRSNDPDDDSECNRTRTDNAVQNRFSCNGAQDIRQWVYIPVEIICVRPPEKKIYGSYPVFNGNLYRRGDIYSPKTYGIDDVLKTDILAKYSRCKDDTDRNSGRVTIQSRGLNYRGTYAEFALMDTRLAMANSMAYVAVKNPEHGVSEVMLSARDSCGRICKAYCKNNAPGSSDYRPCNGIIRVTSRTPKLYGRHYGENVYDMWNLPLGENCPSIKKTQVSVKFFCDFKNEWPWHSDFVLQQRQRGHFHGRRVTGNGPVRQMIMPSMVRGIVQENRIPSLQQEFISGNRKLPGCFLGHNCLVPGPCKPCQNGQKLQCLNTHISFAVCNNGAYVIRQCIGRSHINGHSLMCMGDVFRKPFLYR

> *C. gigas* TyrA6 (complete)

MAIVWTKAVSVFCLVLSLLKLSSALLEPNPFPKTLKECYDFRSYNMTPSDEVALMIQNHCFKNYQYKQIADGKIWTAPNITQEGMNYINSLFRKLFGEIVASSKSKHQKRQAAVRFRREVRSPGAFAPFVECIRRLYNTQVSANMNEYQAIASLHTGQALQSAHDGAAFLPWHRIYLLLLETACRGVPVPYWDSTVDHVMPDPTRSIVWSEQFFGNGDGQVLTGPFRNFQTTVPGDSITREIGTSRNALFTKEGLAAVLSRTRYSEIVEPKRGREYVYSLEGHHNGPHNWVGGHLPLPWVAAFDPVFFMHHAYVDAVWEVFRAQQIRNGIDPGRDYPLQNEPGHGPFDIIDFRPYFPPIRNIDAMSQAVARLVRYEPFPSCQNNCNSSPHLTCVGGVCMSRARPAASPIESFGGAAAFGPSAQDVPSQSRVLAQARGPIPGGERFRSSPFMDTRNRPNTIGNAPVAPELEAASFQARTAGMRSKRDASKILHYNVSSNAHVQSVSSLERSYTNTFIMDGVIDVKRWVYVPVRVVYNRTNANGNDPTFKANILKENLNEMCRAVGSGASKVYVASNGLDYFGTYKEFAIIDERQPISETTAAVGIKNPDYGAGEVMFSAYDSCGRPCRPFCLTSIQGKQKYRPCSGVFKISSAEXQRCVCP

> *C. gigas* TyrA7 (complete)

MPGLLFVSLCVGFLGLSLALLEPIPFPASLQECYEYRSYNMTPSFEAAHQIQQYCYRNFEYQQIASGKVWSGTNITIQGINYIDSLFRQIFREVEEMERQNKNGRRTKRQTIGRRYRREVRSPGAYQPFADCIVRLQNQFVEDPSTGRNTYQTLAAFHSGQALRTAHGGPGFAPWHRIYLLLLETACGAPIPYWESGLDHDMEDPTASILWSDDFFGNGNGVVTTGPFRSMRTILGGPIIRNYGTGEGALFTKTGYNAVLSRTRYDDISEPKQGAAYFFTLEGHHNGPHTWTGGHLARPNSAPYDPVFYMHHSYVDAVYEAFRQRQRQNGINPETDYPVNTPPGHGFDDLIDFRPYINQITNRYAMSDAVANLVTYEPFANCRNRCNGSPHLYCQNGVCVSRNRPTAAPSGTFAFGDSDGAFRRNQERMRFAEAAGPIPAGEKFRTAPLRDIRNQEDRLGIAPVAPEIQAASMQVREVQARFRRDVSHLSKNESLHHGSHQSISSIGRSYTNSFILDGVVDQKRWVYVPVRIVYTRSPNVKGTDPTLLGNSELQNDVCQTAHSGASKVFVASDGLDYYGNYKEFAIIDERQPVSVTTTAVGIKNPEFGEGEVLFTSYDSCGRPCRPLCLTSVNGQQKYKACSGAFKISSAAPTLYRNSYKEAISVSLSSYNLVDSSLDDASPPVTFVCGNDNAWPWVY

> *C. gigas* TyrB1 (complete)

MVDYSSWVISELIQLRLADQESVEHSRAFPDNIVKPSAMSERSRICNERNQLGDKLKNGLDEDALHKMRWASYYAIFLVVLETHAAVWECKLPGLFKECYDFYSRMARVQDTPAYSIQSRCINSYLWKTSFVRYHVSLSTSDINYIRSLQREMESKRRYKRYKRQAATPVAVRREFRTLSDAEREAFFNAVNAMKNDGRYDALANMHTGIALQSGHEGPGFLAWHREYLTAFETALRRVDPSVSLPYWDSSKDFVMDNPALTSFFSSALVGNGDGIVVNGPFAGWPARPDGQRLSRDIGVIGSLFTPEGLDLFLNDPTVNLTRQIVLGTNEMLANTLEGQHNNVHNWVGGDMSRLNTAAHDPVFFMYHAHVDYVWERFREKQIALGSANPEADYPIINSPLHQPDRAMDGFPNVTNLEGFSNRYTSELYTYANSPECADGCGGSRFLRCSTTINRCVALTAAEVGGTADTNQNPVVDMGDFQFFNTFNDPRTRFARSTEHIRHKRDNAYIQTLLPKYALNLSHKPTSIANPFTKYGFNNHKTTITHKPKYASKTAVIHGTTPVLERGRQNLFSLDGVCDVSRWAYIPVKIINERDVSGPGFNSPIIVRDQFIPGVDIYNPLVYTALRPLFYQTRGQVVAKFPTCKKVNSGVKKVYIRSDGLTYDGHFIEHAVMDERQPISETTAYVAVKHPKLGPGKAILTAYDSSGRVCEPKCLIPGSYPPSYRTCSGVINISNLPPKMYGDDYGQAVRSQWNFVPGGCPSQQRSPIYLVFYCSPSVQWPWKKCLT

> *C. gigas* TyrB2 (partial)

MAATCVLKVFLGLVLFAFRTHSIMWEADMPSLLKKCYEEHTRGLTVSDIASHDIQSYCLGSYIWQIPHIQRQVNMTESQINYIKSVYREYQHKMYSARRQKRQAPQRALRRELRVLSDAERQRFFDALNALKADGSYDAWANVHANMMVIRSGHEGPNFLGFHRVYLLFFEFALQRIDSSVSLCYWDSTMDNDMAEPQETAMFTSQLVGNGNGPVVNGPFSNWEEDDGPLTRNIATVRSSLMVKTALNRFFEGNAATTHRDVVVGFGVNLANTIEGQHNNVHNWVGGTMADAVTTAYDPVFILHHTFIDYVWERFRQKIRQQGTDPTDYPWPMQNADWNTNFLHYTNRPMDLYENFTNADGYSDIFTQDFYQYEDMPSCATNCGNSRFLTCQGGVCVGLTASDIGEDSSAQVMGQTAFLGRAAAEMEERPVVAPFQTSFVDPRTGNGNGNGGNLAAPASRSPVGFSPFQSSMNDPRTGNGNGWNGIRQQSRGSPAGSLQGMQRFNAGVGPMQLNRAPIAANLRNPAGIGIRNVGGMFRPNSPNTLARFGSLVTPNNPIRHLWDQSVRRNSFQRSNLARSHLHSSGLLNADDIRRMVHLRDLPIQNTFTMDGVSDARRWVYLPVRIVYMRPPGQFFGSRMVYNSQFVRASDMYAPQIYPEFNNISPKIAPATYPNCLKNLGGSSKVFVQSNGFSYKGKYLDYAVLDERQPVSESIAYVAVKNPDLGAAQCYLTAFDSCGRVCQPRCLIPGSNPPAYRPCSGVVNVSNRLPRMYGRTYGEAVKSRWSFSDKNCPSSFQGEIFMTFYCDYENVWPWKGCNGGTTRRKSYAGWWSIDIK

>*Perna viridis* TyrA1 (complete)

MLKILVCLVALNSFCGVKCLISQIDMPPYLKDCIKLKTSKHDPTQSPSEDVCNTCVTRYVWIKGPNLKKCSHQQDNTTMADISRFFGKVICDHYNYRQKRQARTHMRQEIRMMSPQRRKNLRAAWKKAYDDGYFGWLARFHNDQIRDSAHAGPAFPGYHRFFLLMLESVLKHYDKDITMPYWDSTKEANMENPANSVLWTDDYLGEMNGVVNSGMCGGFRDLRGNPIMRNGGNAGSLFTSYDTRFVINIPNIERLTEPSPYVTLESAHDNVHNWVGGTMAPIELAPWDCIFWMHHAYVDYLWEVWRYNHNYDMKYPYKAGLPGHGPNTPMKNMPYMRFLGRVPTNKDGYSARLAKLAYYQLSPTCSAQNTYCGSPDLQCKIGRYASECVSVDVRFRRETQPFRQANNRGGNVNFKVNRAGRKKRRADHQGYYEDYEFSVPADQIDNIHIPIEREPCMGRPIQNNFIADCSSDAKQWVYLPVKVVHLRPQEVVFKASGHDYGQPSRYDMYDEHNYAKLNQYVKPGNPAVYEDCMEDESGAFKVRLKSSGLSYFGSYTDYVFVDNRLPVSSHIGYIAVQKPTPYKPTDVLITASDACGRLCKATCRKQVGNHVYYEPCKGTIRVTPDMPLMYGNDFGEAVLSIWEFNGKFTPTESEHNIYLEFYCDYSNKWIWDECPHSKQG

>*P. viridis* TyrB1 (complete)

MVEFSAFMPDMNHVLPLLVILLYHQYDVTMSKVISEGFPGEIADCRYVERSELYTCIRSKFDNPDWIPDSGPKLQLRLRQDFYWHPTKRIRRECRAIPREEWHALCDAINKLKKDKTFAPNVYDNFADYHTNEAVNSIHFGPNFFGWHRIFILVFEELLRKVNPNVTLCYWDSRLDHNMKHPEKSIMFTKEFFGNTKGPVVTGPFAGWKTIRNIPLRRNSAQEGELISTAAFDKVLSKHYHVQITTPTADDDSNVESLHNGVHAYIGGQMNDFNTSSQDICFWFHHAFIDSVWEKFCSKLRNKGLDPQEDYVIIDERLHRPRRLMDHFFPFKNIDGYSDYFPRNIYKYEEYATCPDCLNSPYLKCNNVTNKCYSIQENEPKRLVFKPLKKIETNHLSAPKDEFKIISGKENLESMVFVPVKVVFRDAVHKSIRKQYIAGCEYQKKDIDVCKKSVAIVESHGMSYHGKYRNYVINDACVPQWVYAFVGVRDPALGDSMSFISVTDKLGNHCFAYCLDLEAKKYRSCSGVIKLTTDTPRMYSKSLDDAKASGFPFSPLEADTLDPRIQLSFLCY

> *P. viridis* TyrB2 (complete)

MQVKTFVYFYICLSVHLANGNISPDIEQQCKDVQDSHMCFFNLLKNNTEINRTTLIIQEEYFWHPVKRKRKDCRALSEKERNDLFSAINALRKDKSKTPNVYDNYAAFHTQRTLKSVHHGPNFFGWHRIYLLRFEELLRKVNPSVTLCFWESPLNYYMKNPLDFVMFTDDFLGNGRGTVTTGPFANWTTMDNRPLRRRLGKHRDGRLLAPFEIKYVLSKNRHSQITHPNADFGSDLEMMHNVVHILVGGQMGDLKTSTQDPFFWFHHAYLDSVWEQFCAKIRQNGIDPKDDYVKVKNKLHHPKRRMDRLFPFRNEDGYSDYFTKYVYEYDEHAKCPYCSYSPYLDCNKKKNVCYALSSRDKTRNITMPFHDFAPTGPGHKISILSGQENAKRLIFLPIKIIFEDALKKNLHMKKIAGCQFEQRSR

>*Lottia gigantea* TyrA1.2 (complete)

MRIALSLLLLLSIVTDVEPLIREAPLPKQLKECYQKYSRKSLASVVGKSLCWYCETSLRGRMNPPAEPMVLPNRRDYRRLAEPLINRRVKRQAGGSTCIRKEYRMLTSAERDNYHNAINALKQDTTMTPNMYDAVAMFHVGDASVRAHGGPGFLGWHRMYLVMYERALQSKVPGVCIPYIDNTIEAELGDDGSYLWSDEFLGTPNGVVTSGPFANWNTPIGELTRNVGNQAFPMDKDILNDIMSRGRIEDIVSPTAELEHDIEYHHGSYHIHVGGLMESIDTASFDPVFFMHHAYIDYVWEQFRQKTLAAGGDPTRYPESNDLPLHTGDTVINVIQLPTGNVTVTQRDMYATLTDYEYQPSPECSRTNPDCGSIYLACNRTSYRCYPVNPSLQPPVNPGPPINPGPPVNPGPPVNPGPPVNPGPPVNPGPPVDPPTDPWTPVDPRPPVNPGPPINPIPPVNPRPSNCIDSNRYIKAPRTCQNTFCIDGVCDTNKWSYFPVKVISNRAPEFSGSGSYPVRNGKVMTKNDIYEPKAYTAVNQYVRPRAVEPKGYGNCQDTHGIGQIFISSQGINYEGYYSESSIIDQRLATSISIAYLAVKDIGISGKSTALLRAFDQCGRICHTACRVPGSNPAKYRPCSGVVDITPDFPKQYSSSYGESLLQVWGYKQDRECPIFEGEDFFMSFYCDYKDRLPWVESGPVYGQTQPSPLIMPIFQPKPVVPPKPKAVCKVNTKCTVEVACESRKCSTYGHIILETILALGYPRNSV

>*Laternula elliptica* TyrA1 (partial)

MGRLVLLVTQLVLLYMSMSTAQPANQMCLLIPPDIQTCYRAYQNTTISSLAAQVACLKKLLWRFSPDSSLTNEDITNIRTTGGAAGFSASFATSDPFPETGFRTRKEFRSLTTNERTRLFRALRQLHTNGVIRSYARLHLQANRNIHNGAAFLPWHRAFLSYFEEDLRKIDPTVSLPYWDSTIDVEMSSPALSRLWEEGLFANGVGEVVTYPFDRWTTDSGWLERDVGRRGVLISKQNVARVLTKCTLGEISEPTRVQRRDSWEFYHNGVHNFVGGDMVSWLSPYDPVFFFHHAFIDYVWELFRERQRSTCGVDPTTDYPATSGGHGPNSVMPGYPHLRNIDGC
